# Single DNA hairpin nanowire based on self-hybridization chain reaction for sensitive ATP detection

**DOI:** 10.1080/14686996.2026.2619337

**Published:** 2026-01-23

**Authors:** Fengyi Lin, Jing Liu, Yuxin Cheng, Min Li, Hong Zhang, Cuisong Zhou, Yong Guo, Dan Xiao, Peng Mi, Jianyuan Dai

**Affiliations:** a College of Chemistry, Sichuan University, Chengdu, China; b College of Polymer Science and Engineering, and Department of Radiology, Huaxi MR Research Center (HMRRC), and State Key Laboratory of Biotherapy, West China Hospital, Sichuan UniversitySichuan University, Chengdu, China; c Hospital of Chengdu University of Traditional Chinese Medicine, Chengdu, Sichuan, China

**Keywords:** DNA nanowire, self-hybridization chain reaction, biosensor, ATP detection, cell death detection

## Abstract

Hybridization chain reaction (HCR), which typically consists of two hairpins for signal amplification, has emerged as a versatile tool in bioanalytical applications. Here, a novel HCR nanowire based on a single DNA hairpin structure is reported. The hairpin stem is rationally engineered with a palindromic sequence, which enables a self-hybridization chain reaction (SHCR) upon the introduction of the initiator DNA strand. Compared to the conventional two hairpin-based HCR nanowire, the single DNA hairpin-based SHCR nanowire achieves nearly a two-fold improvement in the signal-to-noise ratio and exhibits better selectivity for single-base mismatch. By integrating the initiator DNA strand with adenosine triphosphate (ATP) aptamer, the single DNA hairpin-based nanowire has been applied for sensitive ATP detection, capable of monitoring ATP both in living cells and that released from dead cancer cells post-radiotherapy. The SHCR nanowire we proposed here has significantly simplified the sequence design of HCR and holds promise as a potential alternative to the conventional HCR nanowire.

## IMPACT STATEMENT

This study succeeded in producing single DNA hairpin nanowires based on self-hybridization chain reaction for intracellular ATP monitoring and evaluating the viability of cells in vitro.

## GRAPHICAL ABSTRACT

**Figure:**
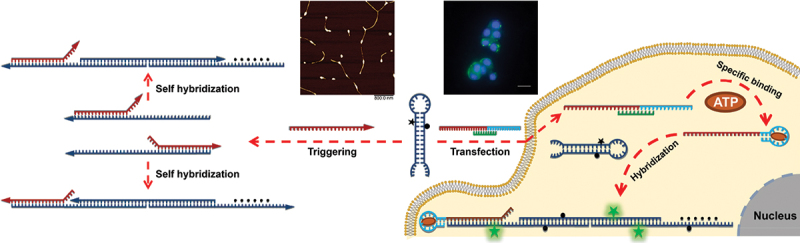

## 1. Introduction

Nucleic acid is one of the most indispensable biomolecules, playing critical roles in biosystems such as cell differentiation [1,2], and the applied life and material base for storing, encoding, and transmitting genetic information [3–5]. Deoxyribonucleic acid (DNA) and ribonucleic acid (RNA) are the two main types of nucleic acid macromolecules, in which DNA can be controllably and accurately assembled to various nanoscale architectures and devices through Watson Crick base pairing principle [6–8]. Enzyme-free DNA circuits, including hybridization chain reaction (HCR) [9,10], catalysed hairpin assembly (CHA) [11,12], and entropy-driven catalysis (EDC) [13,14], are the dynamic nanostructures that can be rationally programmed and operated by the toehold-mediated strand displacement reaction [15,16].

HCR, a classical DNA circuit, was initially described by Pierce in 2004 [9], and contains a pair of kinetically trapped DNA hairpins (H1 and H2) that exist in a metastable state when mixed in solution. Upon the introduction of a single-stranded initiator DNA, H1 is unfolded, revealing a new single-stranded region that proceeds to open H2; then, a sequence region identical to the initiator DNA in H2 becomes exposed. This series of events repeats, ultimately resulting in the formation of a nicked double helix via the chain reaction [17]. This isothermal, enzyme-free polymerization process has been effectively utilized for the signal-amplified detection of both nucleic acids and a range of non-nucleic acid targets [18–23]. In general, the hairpins employed in conventional HCR nanowires are 48 nt (6 nt loop/toehold and 18 nt stem). To reduce the cost and complexity of sequence design, HCR hairpins of 36–42 nt in length were designed, but obvious signal leakage occurs due to the short stem [24,25]. To solve this problem, a peptide nucleic acid (PNA)-based HCR with 20 nt hairpin [26] and *acyclic* d-threoninol nucleic acid (d-*a*TNA)-based HCR with 26 nt hairpins were developed [27]. However, current HCR nanowires still meet some limitations (*e.g*., still complicated), especially, since two hairpins are indispensably required.

Herein, a facile self-hybridization chain reaction (SHCR) nanowire based on only a single hairpin structure (H) with palindromic sequence design in the hairpin stem was developed, and the single DNA hairpin-based SHCR nanowire can be triggered by an initiator DNA (I). For further biosensing applications, the nucleic acid aptamer was introduced into the proposed single DNA hairpin-based SHCR nanowire, while adenosine triphosphate (ATP) was selected as a model molecule to initiate the chain reaction. Finally, a simple and sensitive single DNA hairpin-based SHCR nanowire for ATP detection in vitro was successfully achieved, demonstrating high potential for bioimaging and monitoring cell death.

## 2. Experimental section

### 2.1. Materials and reagents

Tris(hydroxymethyl)aminomethane (Tris), magnesium chloride, sodium chloride, adenosine triphosphate (ATP), guanosine triphosphate (GTP), cytidine triphosphate (CTP), uridine triphosphate (UTP) and RMPI-1640 medium were purchased from Sigma-Aldrich (St. Louis, MO). DNA oligonucleotide sequences were synthesized by Sangon Bio-technology Co., Ltd. (Shanghai, China) and purified by high-performance liquid chromatography (HPLC). The sequences of oligonucleotides were listed in Table S1. Lipofectamine 3,000 and Hoechst 33,342 were bought from Invitrogen (Carlsbad, CA). Mouse breast cancer cell line (4T1) and human in situ pancreatic carcinoma cell line (BxPC-3) were bought from Procell Life Science (Wuhan, China). All other reagents were of analytical grade and were used without further purification. Ultrapure water obtained from a Millipore water purification system (≥18.2 M Ohm cm) was used in all experiments.

### 2.2. Instrumentations

Gel electrophoresis images were scanned by the digital camera of a UV imaging system (Clinx Genosens, China). Atomic force microscopy (AFM) images were recorded with a Bruker Dimension® Icon™ AFM in tapping mode under ambient air conditions. All fluorescence measurements were performed using a F-7000 fluorescence spectrophotometer (Hitachi Co. Ltd., Japan) and an Infinite® 200 PRO multimode plate reader (Tecan Group Ltd., Switzerland). Cells were irradiated within RS2000 Pro X-ray irradiation apparatus (Rad Source, U.S.A.). Fluorescence microscope images were obtained on BZ-X810 (Keyence, China).

### 2.3. Secondary structure simulation and tertiary structure prediction

UNAfold [28] was utilized to predict the secondary structures of sequences, which were uploaded to 3dRNA/DNA web server [29] and acquire the DNA tertiary structures. Then, energy minimization and visualization were carried out on three-dimensional DNA structures by the PyMOL.

### 2.4. DNA assay

H and I were annealed for 5 min at 95°C in Tris buffer (20 mM Tris, 140 mM NaCl, 5 mM MgCl_2_, pH 7.5), respectively, and cooled slowly to room temperature for 2 h. For the DNA assay, 500 nM H and different concentrations of I were mixed in Tris buffer to achieve a final H concentration of 300 nM. The obtained solutions were incubated at room temperature for 24 h, and followed by electrophoresis experiments or fluorescence measurements.

### 2.5. ATP assay

A pretreated H hairpin probe was prepared as described above. The trigger-aptamer (TA, 1 μM) and blocking probe (BP, 3 μM) were mixed together in Tris buffer and annealed for 5 min at 95°C to form the TA-BP duplexes. The one-pot method was used to obtain ATP assay by mixing different concentrations of ATP with TA-BP and H in Tris buffer, where the final concentration of H was 300 nM and that of TA was 100 nM. After incubation at room temperature for 3 h, the solutions were characterized by a F-7000 fluorescence spectrophotometer with a 2 mm × 10 mm quartz cell containing 100 μL solution. According to the fluorescent properties of FAM, the excitation wavelength was set to 487 nm; slit width for both excitation and emission was set at 5 nm.

### 2.6. Native PAGE analysis

Loading buffer was added to each sample and 5 μL each of them was loaded into the lanes of the freshly prepared 12% polyacrylamide gel. The gel was run with 1 × TBE buffer (45 mM Tris, 1.0 mM Na2EDTA, pH 8.0) at a 100 V constant voltage for 1 h at room temperature. Ethidium bromide (EB) diluent was used to soak the gel to image each sample. Finally, the polyacrylamide gel electrophoresis (PAGE) images were scanned by the UV imaging analysis system. The gray values of the bands in the gel electrophoresis image were measured by ImageJ and the relative yields of SHCR products were calculated using the following formulas (1) and (2):

(1) $$\mathrm{RelativeyieldofSHCRproduct}\;\mathrm{left}(\mathrm{includingby}-\mathrm{product}=\frac{\left(\mathrm{GrayvalueofSHCRproductinlanen}\right)-\left(\mathrm{GrayvalueofSHCRproductinlane}1\right)}{\left(\mathrm{Total}\;\mathrm{gray}\;\mathrm{value}\;\mathrm{of}\;\mathrm{the}\;\mathrm{SHCR}\;\mathrm{system}\right)}\times 100\%$$

(2) $$\begin{array}{rl} & \mathrm{Relative}\;\mathrm{yield}\;\mathrm{of}\;\mathrm{SHCR}\;\mathrm{product}(\mathrm{without}\;\mathrm{by}-\mathrm{product})\\ & \;\;\;\;\;\;\;\;\;\;\;\;\;\;\;\;\;\;\;\;=\frac{\begin{array}{c} \left(\mathrm{Gray}\;\mathrm{value}\;\mathrm{of}\;\mathrm{SHCR}\;\mathrm{product}\;\mathrm{in}\;\mathrm{lane}\;n\right)\\ -\left(\mathrm{Gray}\;\mathrm{value}\;\mathrm{of}\;\mathrm{SHCR}\;\mathrm{product}\;\mathrm{in}\;\mathrm{lane}\text{ 1}\right) \end{array}}{\begin{array}{c} \left(\mathrm{Total}\;\mathrm{gray}\;\mathrm{value}\;\mathrm{of}\;\mathrm{the}\;\mathrm{SHCR}\;\mathrm{system}\right)\\ -\left(\mathrm{Gray}\;\mathrm{value}\;\mathrm{of}\;H\;\mathrm{by}\;-\;\mathrm{product}\right)\times 100\% \end{array}} \end{array}$$

### 2.7. AFM imaging

The prepared hairpin probe was mixed with annealed I, followed by incubation at room temperature for 24 h. The reaction solutions were placed onto two pieces of freshly cut mica, dried in the air, and lightly rinsed with double-distilled water. The excess water was removed with filter paper, and the samples were left to air-dry again before AFM imaging.

### 2.8. Comparison of SHCR and HCR

The modified H and bare H were mixed, meanwhile, H1 and H2 were also mixed, making their final concentration 250 nM. After annealed according to the above process, mix the SHCR hairpin with I, and the HCR hairpins with IHCR, ensuring the final concentration of all hairpins was 150 nM, and that of initiators was 50 nM. Finally, the change of fluorescence intensity of each system with time was recorded.

### 2.9. Kinetic analysis

Firstly, background fluorescence value (*F*_*0*_) and relative fluorescence intensities (*F/F*_*0*_) of HCR and SHCR were normalized to the interval [0,1] respectively. To determine the maximal rate (*V*_*max*_), we obtained a smooth fitting of normalized results using a 5-parameter asymmetric sigmoidal formula (Logistic5). There is no physical meaning for this formula, just to obtain the best fitting. Once obtaining the best fitting, we then determined reaction rate at each time point by calculating the slope of tangent at each time point. Vmax is the peak of the corresponding reaction rate curve, indicating the maximum value. According to the three-step reaction mechanism proposed by Zhang and Winfree [30], HCR and SHCR can be simplified to the same reaction model as follows:

(3) $$\mathrm{Reaction}1:I+H^{5{\;}^{\prime }}-H^{3{\;}^{\prime }}\begin{array}{c} k_{\mathrm{on},\mathrm{Toe}}\\ ⇌\\ k_{\mathrm{off},\mathrm{Toe}} \end{array}I-H^{5{\;}^{\prime }}-H^{3{\;}^{\prime }}$$

(4) $$\mathrm{Reaction}2:I-H^{5{\;}^{\prime }}-H^{3{\;}^{\prime }}\overset{k_{b}}{\to }I-H^{5{\;}^{\prime }}+H^{3{\;}^{\prime }}$$

where $H^{5{\;}^{\prime }}$ and $H^{3{\;}^{\prime }}$ represent half of the hairpin respectively. Leakage rate constant ($k_{l}$), forward rate constant ($k_{\mathrm{on},\mathrm{Toe}}$), reverse rate constant ($k_{\mathrm{off},\mathrm{Toe}}$) and branch migration ($k_{b}$) were calculated by the following three formulas:

(5) $$k_{l}=\frac{V_{0}}{{\left[H_{1}\right]}_{0}{\left[H_{2}\right]}_{0}}$$

(6) $$k_{\mathrm{on},\mathrm{Toe}}=\frac{V_{0}}{{\left[T\right]}_{0}{\left[H\right]}_{0}}$$

(7) $$k_{\mathrm{off},\mathrm{Toe}}=\frac{2}{\mathrm{length}\;\mathrm{of}\;\mathrm{toehold}}\cdot k_{\mathrm{on},\mathrm{Toe}}\cdot e^{\frac{\Delta G_{\mathrm{toehold}}}{\mathrm{RT}}}$$

(8) $$k_{b}\approx \frac{400}{{\left(\mathrm{length}\;\mathrm{of}\;\mathrm{branch}\;\mathrm{domain}\right)}^{2}}$$

where the value of $\Delta G_{\mathrm{toehold}}$ is derived from the toehold binding energies reported by Zhang and Winfree.

### 2.10. Cell culture

Both 4T1 breast cancer cell and BxPC-3 pancreatic cancer cell were cultured in RMPI-1640 medium containing 10% fetal bovine serum at 37°C with 5% CO_2_ supply.

### 2.11. Cell viability assay

The cell viability was evaluated by CCK-8 assay. Firstly, 4T1 cancer cells were seeded into 96-well plates (10^4^ cells/well) and incubated overnight. And then 50 μL of the SHCR nanowire with different concentration was added to 4T1 cells containing 50 μL RMPI-1640 medium. After incubating for 24 h, 10 μL of CCK-8 solution was added to each sample, followed by incubation for 2 hours and detection of the absorbance at 450 nm. The cell viability was calculated by the following formula:

(9) $$\mathrm{Cellviability}=\frac{\mathrm{Ab}s_{\mathrm{Treated}}-\mathrm{Ab}s_{\mathrm{Blank}}}{\mathrm{Ab}s_{\mathrm{Control}}-\mathrm{Ab}s_{\mathrm{Blank}}}\times 100\%$$

where $\mathrm{Ab}s_{\mathrm{Treated}}$ represents absorbance of the experimental group, $\mathrm{Ab}s_{\mathrm{Blank}}$ represents absorbance of the blank group, and $\mathrm{Ab}s_{\mathrm{Control}}$ indicates absorbance of the control group.

### 2.12. Detection of ATP in cells

The 4T1 cancer cells (1 mL) were placed in the confocal dishes (30 mm in diameter) for 12 h. The SHCR nanowire (H: 300 nM, TA-BP: 100 nM) dispersed in 800 µL RMPI-1640 medium was mixed with 200 µL RMPI-1640 containing 5 µL lipofectamine 3000, and incubated for 10 min at room temperature. Then, the mixture solution was added into cells to incubate at 37°C for 24 h. After removing the SHCR nanowire medium and staining the nuclei with Hoechst, the 4T1 cells were washed with PBS for three times and imaged by a confocal laser scanning microscope (CLSM). The fluorescence of FAM was excited at 488 nm and collected the emission from 500 to 550 nm, and the fluorescence of Hoechst was excited at 405 nm and collected the emission from 425 to 480 nm. For monitoring the influence of drugs on the intracellular ATP levels, the 4T1 cells were pretreated with Ca^2+^ (5 mM) or oligomycin (300 nM) for 30 min, and then the intracellular ATP was monitored by above processes. The fluorescence intensity was quantitatively monitored by the CLSM software and compared.

### 2.13. Detection of ATP in media released by X-ray-irradiated cancer cells

The 4T1 breast cancer cell and BxPC-3 pancreatic cancer cell were irradiated by X-ray at the dose of 8 Gy, respectively, and then incubated at 37°C for 24 h. Cancer cells without X-ray irradiation (0 Gy) were applied as controls. The ATP in the supernatant (5 μL) was detected by adding the SHCR nanowire (95 μL) and incubating at room temperature for 3 h, followed by measuring the fluorescence intensity using a multimode plate reader. According to the above method, the ATP concentration-response curve was generated by a series of ATP with known concentrations.

## 3. Results and discussion

### 3.1. Rational design of the SHCR nanowire

**Figure 1.**
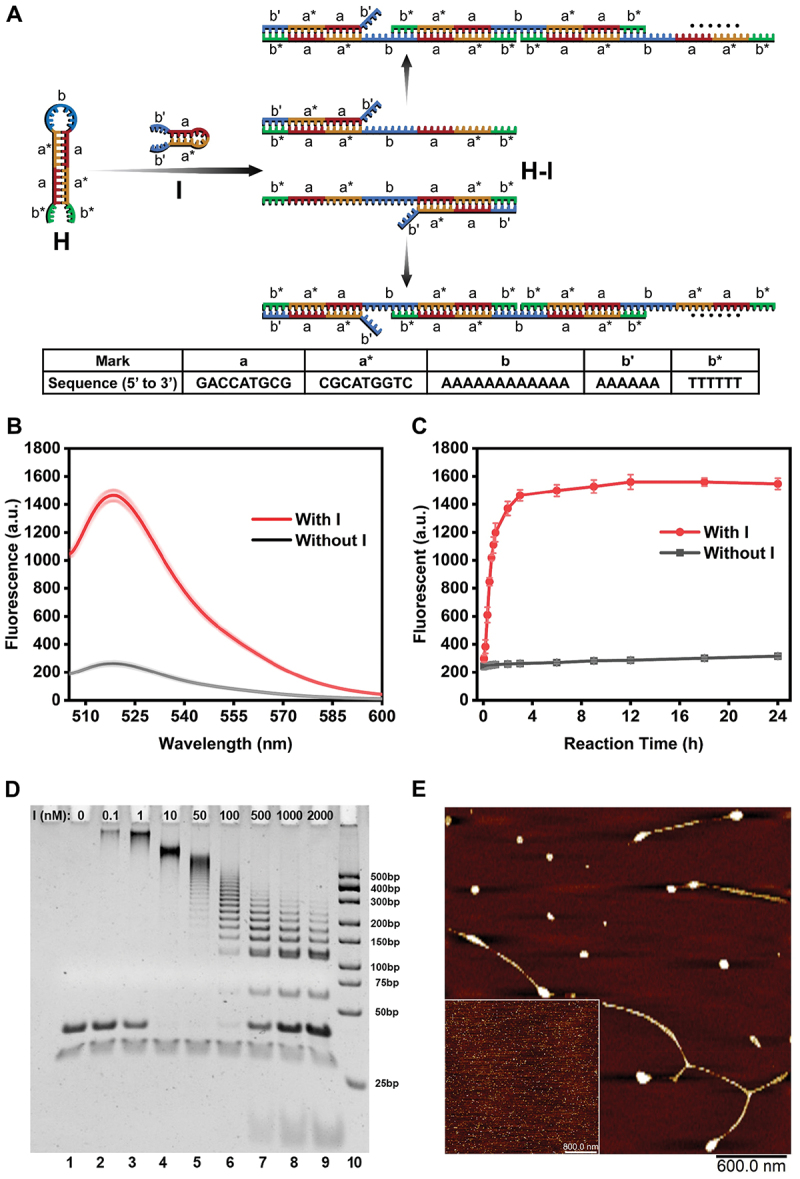
Characterization of the single DNA hairpin-based SHCR nanowire. (A) Schematic illustration of developing self-hybridization chain reactions with one hairpin. (B) Fluorescence intensity and (C) Time-response curves of the SHCR nanowire with and without 50 nM I. H concentration: 300 nM. (D) Gel electrophoresis image of the SHCR nanowires. Lane 1–9: nine different concentrations of I (0, 0.1, 1, 10, 50, 100, 500, 1000 and 2000 nM) reacting with 300 nM H; lane 10: DNA ladder. (E) AFM images of the SHCR nanowires in the presence and absence (inset) of I. H concentration: 300 nM; I concentration: 50 nM.

The development of the single DNA hairpin-based SHCR nanowire was illustrated in Figure 1(A), while the DNA sequences were listed in Table S1. The stem of H was engineered with super palindromic sequences, while the loop and two toeholds of H were engineered with twelve adenine (A) bases and six thymine (T) bases, respectively (Figure S1A). The design of super palindromic sequences not only ensures the formation of a stable hairpin structure but also facilitates the hybridization between two H. In order to improve the hybridization efficiency between I and H, six A bases were placed at both ends of I, and then H can be opened simultaneously by I through 5’-toehold and 3’-toehold to form the intermediate product H-I. Subsequently, a sequence region analogous to I became exposed, enabling this exposed sequence to unfold another H to repeat the chain reaction. Finally, nicked double helixes or DNA nanowires were produced.

### 3.2. Validation and characterization of the SHCR nanowire

Fluorescence spectroscopy, gel electrophoresis, and atomic force microscopy (AFM) imaging were applied to evaluate the single DNA hairpin-based SHCR nanowire. For fluorescence measurement, a fluorophore (FAM) and a quencher (BHQ1) were labelled to the stem of H, and the fluorescence of FAM was quenched by BHQ1. As shown in Figure 1(B), a weak fluorescence signal was observed in the system without I, while the single DNA hairpin-based SHCR nanowire exhibited a significant enhancement of fluorescence signal when I was added, indicating that the SHCR reaction was triggered by I, and FAM and BHQ1 were separated during the formation of DNA nanowires. Furthermore, the response curves over time showed that the untriggered SHCR nanowire exhibited good metastability, while the fluorescence intensity of the triggered SHCR nanowire increased significantly and reached a plateau after 3 h (Figure 1(C)). The gel electrophoresis was further applied to validate the feasibility of the single DNA hairpin-based SHCR nanowire (Figure 1(D)). By introducing I, different lengths of DNA nanowires can be clearly observed in the gel, and the average molecular weights of the products were inversely related to the concentration of I, which was similar to conventional HCR [9]. The light band in lane 1 was the by-product of H after annealing, which was the double hairpin structure (Figure S1(B)) that cannot hybridize with I. Accordingly, quantifying the gray value can also prove that this band almost did not change with increased I concentration (Figure S2(A), Table S2). The dark band in lane 1 was considered an H dimer (2 H, Figure S1(C)). The colour of this band gradually turned lighter with increased I concentration from lane 2 to lane 5. Surprisingly, when the concentration of I exceeds 100 nM, the band in this position became darker (lanes 6–9). This phenomenon can be ascribed to the formation of H-2I at high I concentration (Figure S1(D)). Since the base numbers of both 2 H and H-2I are equal to 120, the bands of 2 H and H-2I appeared at the same position. As shown in Figure S2(B), it can be found that the relative yield of SHCR product began to decline after the excess of I, which indirectly proved that some H were involved in the formation of H-2I rather than forming SHCR product. Atomic force microscopy (AFM) and cross-section analysis were utilized to confirm the hypothetical morphology of the products of the single DNA hairpin-based SHCR nanowire. As shown in Figure 1(E), the SHCR products were DNA nanowires with a height of around 1.7 nm (Figure S3), which was similar to previous reports [31–33]. In addition, nodular structures of the DNA nanowires were observed by AFM, prompting speculation that these structures may be attributed to the overlapping of the DNA nanowires during deposition on the mica surface before AFM imaging. Similar nodular structures have also been found in previously reported DNA polymer chains and DNA nanowires [31,34].

Notably, the hybridization rate between H and I is closely related to the toehold length in the toehold-mediated strand displacement reaction [16]. In the SHCR nanowire, the relative fluorescence intensity (*F/F*_*0*_, where *F* and *F*_*0*_ correspond to the fluorescence intensity of the SHCR nanowire in the presence and absence of I, respectively) was significantly decreased, associating with the reduced number of A bases at one side of I (Figure S4), confirming that engineering six A bases on both sides of I was essential to enhance the toehold binding strength between H and I. To sum up, the significances of designing poly T and poly A on H and I included: 1) reducing the collision probability between hairpins and weakening background leakage due to the poor thermodynamic stability of A or T bases; 2) increasing the combination probability between I and H to a certain extent by adding poly A on both sides of I [35]. Another critical point to note is that an excessively high concentration of H could readily induce premature self-hybridization in the annealing process [36,37], leading to elevated background fluorescence (Figure S5). Therefore, a relatively low concentration of H (500 nM) was chosen in the annealing process to examine the function of the SHCR nanowire. Additionally, the reaction temperature of the SHCR nanowire was increased to the physiological temperature (37°C). As shown in Figure S6, despite a relatively lower reaction efficiency at 37°C compared to room temperature, a quantity of DNA nanowires was still generated after a sufficient reaction time, demonstrating the feasibility of using the SHCR nanowire for subsequent intracellular detection.

### 3.3. Comparison of SHCR and HCR

**Figure 2.**
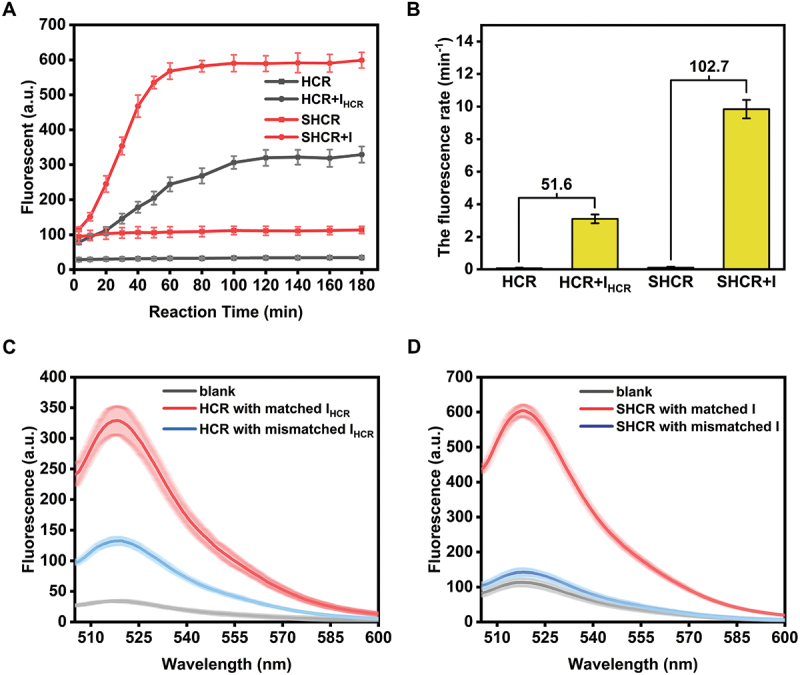
(A) Time-dependent responsive curves of HCR and SHCR nanowires with and without initiator. H1, H2, H, and bare H concentration: 150 nM. Concentrations of IHCR and I: 50 nM. (B) Fluorescence enhancement rates of the HCR and SHCR nanowires were calculated from the linear portion (10 ~ 50 min) of each time-response curve in Figure 2(A). The numbers at the top of the columns represent signal-to-noise ratios of these two systems. Specificity of (C) HCR and (D) SHCR nanowires for matched and single-base mismatched initiators. Reaction time: 3 h. Concentrations of matched and single-base mismatched initiators: 50 nM.

To compare SHCR with HCR, a conventional HCR nanowire was used, in which sequence entirely originated from Pierce’s design [9], and the FAM and BHQ1 were modified on the stem of H1. Furthermore, considering that FAM concentrations in SHCR and HCR need to be equal, half of H in the SHCR nanowire was not modified by FAM, namely bare H. Then, the time-response curves of HCR and SHCR were measured (Figure 2(A)), which can be applied to calculate the signal-to-noise ratio based on the linear portion of each time-response curve [38]. The fitting curve and background leakage rate curve of the normalized fluorescence values of HCR and SHCR (Figure S7A), and the fitting curve and reaction rate curve of the normalized *F/F*_*0*_ of HCR and SHCR (Figure S7B) were obtained based on Figure 2(A). These curves have been used to calculate the maximum background leakage rate (*V*_*l,max*_), the maximum reaction rate (*V*_*r,max*_) and relevant kinetic parameters according to the Li group reported method (Table S3) [39]. Although *V*_*r,max*_ of SHCR was lower than that of HCR, the signal-to-noise ratio of SHCR yielded a nearly 100% increase compared to that of HCR (Figure 2(B)). The standard free energy variation (Δ*G*) for each reaction process of SHCR and HCR was also calculated (Table S4) [40], and the results showed that the background reactions of both SHCR and HCR were weakly spontaneous. In contrast, the initiator-triggered reactions and circuit reactions were intensely spontaneous. Moreover, the Δ*G* values for each stage of SHCR and HCR can be correlated with the reaction rates (Figure S8). Selectivity is another critical parameter of a sensing system. Therefore, the discrimination between matched and single-base mismatched initiators was investigated by using the SHCR and HCR nanowires, respectively. As shown in Figure 2(C,D), the SHCR nanowire exhibited higher single-base selectivity than the HCR nanowire.

### 3.4. Application and in vitro evaluation of the SHCR nanowire for ATP-activated detection

**Figure 3.**
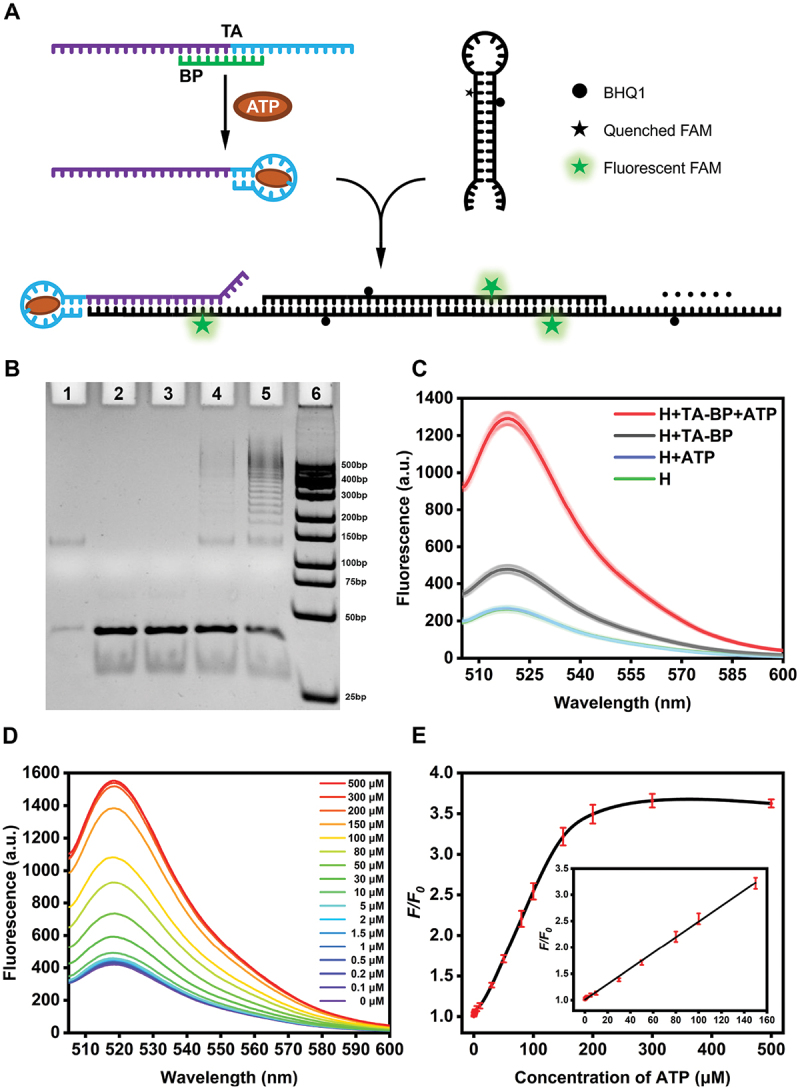
(A) The mechanism of single hairpin-based SHCR for ATP-activated detection. (B) Gel electrophoresis image of the SHCR nanowires in the presence of ATP. Lane 1: TA-BP; lane 2: H; lane 3: H+ATP; lane 4: H+TA-BP; lane 5: H+TA-BP+ATP; lane 6: DNA ladder. The concentrations of TA-BP, H and ATP were 100 nM, 300 nM and 100 μM, respectively; reaction time: 3 h. (C) Fluorescence spectra of the SHCR nanowire in the absence/presence of 100 μM ATP. H concentration: 300 nM. TA-BP concentration: 100 nM. (D) Fluorescence spectra of the SHCR nanowire in the presence of different ATP concentrations (from bottom to top: 0, 0.1, 0.2, 0.5, 1.0, 1.5, 2.0, 5.0, 10, 30, 50, 80, 100, 150, 200, 300 and 500 μM). (E) Relative fluorescence intensity (*F/F_0_*) at different ATP concentrations. The inset shows the linear range of ATP from 0 to 150 μM.

To further validate the application of the single hairpin-based SHCR nanowire, a nucleic acid aptamer has been introduced for non-nucleic acid target detection. ATP is an indispensable energy-providing molecule synthesized in cells for maintaining cell survival and supporting their biological activities [41]. Abnormal ATP level will lead to various diseases [42,43]. ATP also participates in various biological processes of tumor cells [44] and has been regarded as a biomarker of immunogenic cell death [45,46]. Developing sensitive and selective ATP assays is highly required for biochemical studies and clinical diagnosis. Therefore, we chose ATP as a model molecule to validate the versatility of the SHCR nanowire. As shown in Figure 3(A), trigger-aptamer (TA), which contains trigger DNA and ATP aptamer sequences, was designed to hybridize partially with the blocking probe (BP). In the absence of ATP, no SHCR reaction occurred since the trigger DNA was blocked by BP. Once ATP was added, the aptamer was recognized by ATP, and then the trigger DNA was exposed to activate the SHCR reaction. In the gel electrophoresis image (Figure 3(B)), the SHCR nanowire in the absence of ATP (lane 4) only generated trace amounts of DNA nanowires, while the DNA nanowires with different lengths were generated after the addition of ATP (lane 5). The fluorescence spectra (Figure 3(C)) also confirmed the gel results and an enhanced fluorescence intensity was observed when ATP was added compared to the system without ATP. Therefore, gel electrophoresis image and fluorescence signal enhancement proved that an in vitro ATP assay based on SHCR has been successfully constructed.

A series of experimental conditions, including the ratio of BP to TA (BP/TA), length of BP, reaction time, and concentrations of TA, H, and Mg^2+^, were optimized to achieve the best assay performance, as determined by the maximum signal-to-noise ratio achieved upon ATP activation (Figure S9). Under optimized experimental conditions, the sensitivity of our proposed approach for ATP detection was examined. As shown in Figure 3(D), the fluorescence intensity was gradually enhanced with increased ATP concentration, indicating that SHCR was triggered by ATP and more DNA hairpins were opened at higher concentrations of ATP. The calibration curve for ATP determination at various concentrations was shown in Figure 3(E), revealing a linear dependence of the relative fluorescence intensity (*F/F*_*0*_, where *F* and *F*_*0*_ are the corresponding fluorescence intensity of the SHCR nanowire in the presence and absence of ATP, respectively) on the ATP concentration from 0 to 150 μM (y = 0.01478x + 1.01265, R^2^ = 0.998). The detection limit was calculated to be 0.368 μM according to the calculation formula σ = 3S/N (S was the standard deviation of the background fluorescence intensity, N was the linear slope), which was superior to the previously reported ATP assays (Table S5).

To validate the selectivity for ATP-activated detection, several other ATP analogous molecules, such as CTP, GTP and UTP, were examined as controls. The results showed that only ATP could trigger the SHCR nanowire and exhibited a strong fluorescence signal (Figure S10(A)), which implied that the strategy we proposed here has high ATP specificity. To determine whether mutated nucleotides in aptamer could lead to malfunction and further validate specific binding, TA was designed with one or two mutated mononucleotides at positions 49 and 51 to obtain TA_1 M_ and TA_2 M_. The mutated nucleotides made the ATP-binding function of aptamer impossible, consequently failing to expose trigger DNA for SHCR initiation. There was no fluorescence intensity change in response to the interaction between ATP and mutated aptamer (Figure S10(B)), indicating the mutated aptamer could not combine with ATP. Based on the above experiments, the SHCR nanowire was demonstrated to effectively detect non-nucleic acid targets through the introduction of aptamers. It is evident that by changing the sequence of aptamer in TA and the corresponding sequence of BP, the SHCR nanowire can be applied to the detection of other non-nucleic acid targets.

### 3.5. Imaging ATP in living cells using SHCR

**Figure 4.**
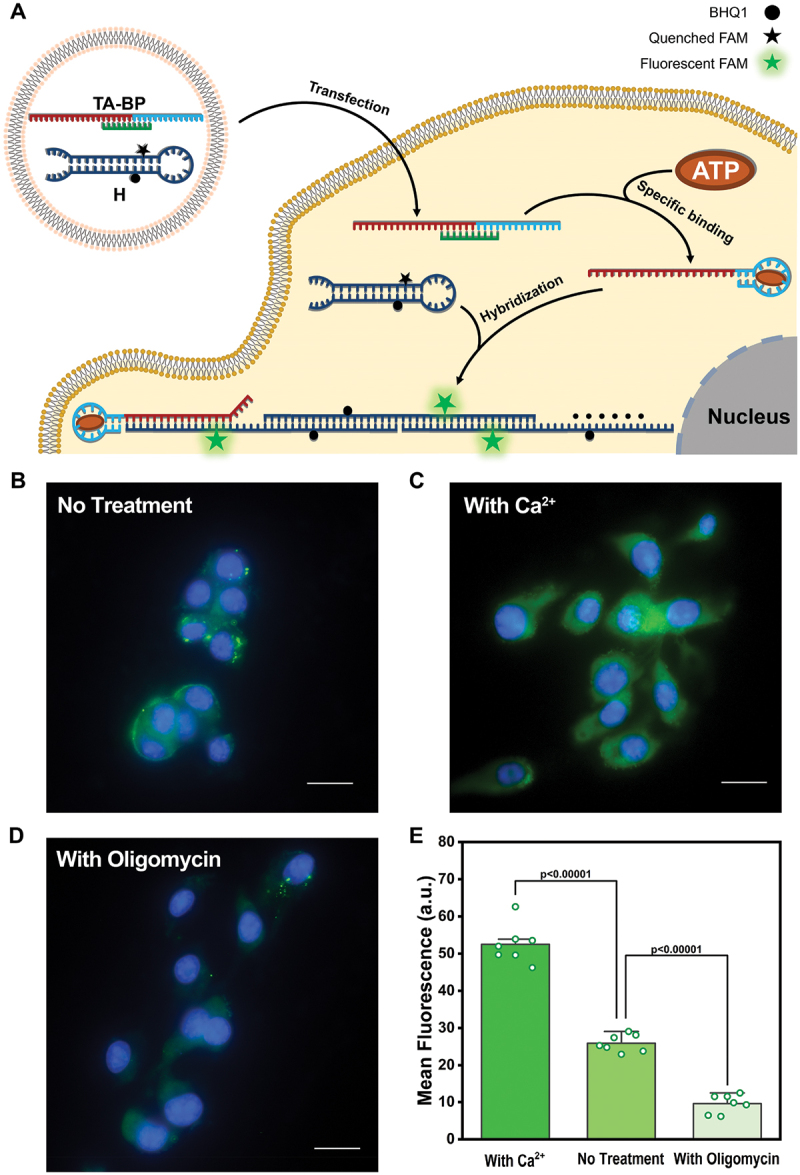
(A) Principle of SHCR nanowires for intracellular ATP detection. CLSM images of 4T1 cells that were (B) untreated and treated with (C) 5 mM Ca^2+^ or (D) 300 nM oligomycin. (E) Statistical analysis of the mean fluorescence intensity. Data in (E) are shown as mean values ± s.D. (*n* = 7). *p*-values were determined by one-way ANOVA with Tukey’s post hoc test. The nuclei were stained by Hoechst (blue fluorescence). The green fluorescence was from the SHCR nanowire. All scale bars are 20 μm.

For normal cells, the intracellular ATP concentration ranges from 1 to 10 mM [47,48], and the extracellular ATP concentration is only about 10 nM [49]. Nevertheless, cancer cells have higher intracellular ATP levels compared to normal cells due to the Warburg effect [50,51]. Considering the vital functions of ATP, we further engineered SHCR nanowire for monitoring ATP in cancer cells (Figure 4(A)). The preliminary experiment, cell viability assay, has demonstrated that the viability of the SHCR nanowire-treated 4T1 cells remained above 80% even in a high concentration of H (400 nM) after 24 h incubation (Figure S11), indicating the favorable biosafety of the SHCR nanowire. This result strongly supported that the proposed single hairpin-based SHCR nanowire was an effective ATP imaging platform. The DNA probes, H and TA-BP, were transfected into mouse breast cancer cells (4T1) via lipofectamine 3000 and incubated at 37°C for 3 h. The fluorescence image (Figure 4(B)) showed that the DNA probe was successfully transfected into the 4T1 cancer cells and captured ATP in the cytoplasm to activate SHCR, producing conspicuous fluorescence signals. Then, the three defective systems (TA_1 M_-BP+H, TA_2 M_-BP+H, and H) were transfected into 4T1 cells under identical conditions to validate the specific binding (Figure S12). These three systems produced shallow fluorescence signals inside cells (Figure S13), indicating that the SHCR nanowire had the potential for sensitively and selectively probing cancer cells through ATP-activated fluorescence ‘ON’, while ruling out alternative explanations for fluorescence changes, such as the release of individual FAM upon the nuclease degradation of the SHCR product. In addition, the SHCR nanowire was further applied to probe and distinguish between the different levels of ATP inside cells. To achieve dynamic control over ATP expression, the 4T1 cancer cells were pretreated with 5 mM of Ca^2+^, a known ATP inducer, by activating dehydrogenases [52,53], or 300 nM of oligomycin, a commonly used ATP inhibitor through inhibiting ATP synthesis, respectively [54,55]. As shown in Figure 4(C), Ca^2+^-treated cells displayed significantly enhanced fluorescence compared to the untreated cells, suggesting that dehydrogenase enzymes were effectively activated by Ca^2+^ in mitochondria and increased ATP production. On the contrary, oligomycin-treated cells showed weak fluorescence (Figure 4(D)), due to the decreased production of ATP inside the cells. The fluorescence signals in different stimulated cells were also compared (Figure 4(E), Figure S14). The Ca^2+^-treated 4T1 cancer cells generated over two-fold higher fluorescence intensity than those untreated cells. However, the oligomycin-treated 4T1 cancer cells only exhibited one-third of the fluorescence signal intensity of the normal cells. Taken together, the changes in the amount of intracellular ATP induced and inhibited by drugs, such as Ca^2+^ and oligomycin, can be detected by the SHCR nanowire in living cells.

### 3.6. Evaluating the cell viability using ATP-activated SHCR

**Figure 5.**
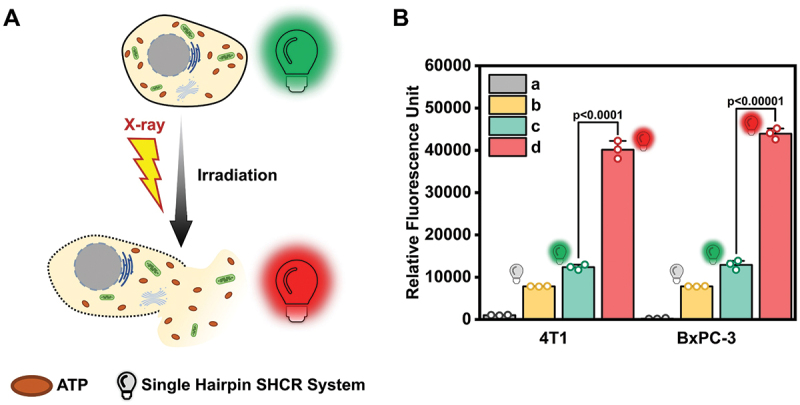
(A) Scheme for distinguishing between living and dead cells by the SHCR nanowire. (B) Fluorescence intensity before and after radiotherapy with X-ray irradiation: (a) cell+X-ray, (b) SHCR, (c) cell+SHCR, and (d) cell+X-ray+SHCR. Data in (B) are shown as mean values ± s.D. (*n* = 3). *p*-values were determined by two-tailed unpaired Student’s *t* test.

Studies have shown that ATP can be released to the extracellular environment from damaged, dying and apoptotic cells [56], and the extracellular ATP concentration can reach hundreds of micromolar due to the release of ATP through the dead cancer cells [57]. In this case, measuring the ATP concentration in the extracellular medium can reflect either the damage or death of those cells. It is known that radiation exposure (*i.e*., radiotherapy) can cause cell death. Therefore, 4T1 breast cancer cells and human pancreatic adenocarcinoma (BxPC-3) cells were chosen as target cells and irradiated by X-ray (8 Gy). The fluorescence intensity of the cell supernatant was then measured by using the SHCR nanowire (Figure 5). The result showed the X-ray irradiation-exposed cancer cells exhibited a nearly 4-fold increase in fluorescence intensity compared to untreated 4T1 and BxPC-3 cancer cells. The fluorescence intensity of the different ATP concentrations in the RMPI-1640 medium was measured using the SHCR nanowire, and the ATP concentration-response curve was obtained (Figure S15). According to this curve, ATP concentration in untreated cell supernatant was calculated to be micromolar level, whereas it reached the millimolar level after X-ray irradiation (Table S6), which is similar to the previous reports [47,57]. This 100-fold difference in ATP concentration before and after X-ray irradiation indicated that cell death caused a greater release of ATP.

To further validate the accuracy of ATP-activated SHCR nanowires for evaluating cell viability, a CCK-8 assay was performed to conduct parallel evaluations of the viability of 4T1 and BxPC-3 cells before and after X-ray irradiation. The results indicated a significant reduction in the viability of both cell lines following irradiation (Figure S16). The absorbance signal of post-irradiation was reduced to 37% of the pre-irradiation level, with average viabilities of 4T1 and BxPC-3 cells reaching 31.1% and 32.5%, respectively. The trend in cell viability changes was entirely consistent with the difference of ATP release detected by SHCR, confirming the reliability and accuracy of ATP-activated SHCR nanowires for assessing cell viability.

## 4. Conclusions

In conclusion, we have successfully developed a facile strategy for hybridization chain reaction based on a single DNA hairpin structure. Compared to the conventional HCR nanowire generated with two hairpins, the SHCR nanowire only consists of a single DNA hairpin structure with a palindromic sequence. This innovative simplification would significantly reduce the cost and the complexity of sequence design and operation. More importantly, the SHCR nanowire exhibits a higher signal-to-noise ratio and single-base mismatch selectivity compared to the HCR nanowire. We have used the SHCR nanowire to construct an ATP-sensing fluorescent platform. The SHCR nanowire could be applied to selectively and sensitively visualize cancer cells through ATP-triggered probe ‘ON’, and evaluate cell viability. Since the aptamer and blocking probe sequences used in this strategy can be changed flexibly, detecting different targets can be easily achieved by simply modifying their sequences, or even by adapting the two into appropriate enzyme strands and substrate strands to form DNAzymes. Importantly, the success of in vitro cell experiments will prompt further investigation into the potential application of the SHCR nanowires for the early in vivo detection of cancer biomarkers. Moreover, since the SHCR nanowire possesses the ability of modularization and scalability, various other nucleic acid signal amplification circuits could also be smoothly installed upstream or downstream of SHCR to develop more cascaded DNA circuits with multiple signal amplifications, accelerating the reaction speed and improving the diagnostic sensitivity. Therefore, the SHCR nanowires may potentially become a lower-cost and simpler alternatives to the HCR nanowires for molecular detection and cell monitoring.

## Supplementary Material

Supplemental Material
