# Supplementary material for: Single DNA hairpin nanowire based on self-hybridization chain reaction for sensitive ATP detection
Source: Sci Technol Adv Mater. 2026 Jan 23;27(1):2619337. doi: 10.1080/14686996.2026.2619337 (PMC12978176; doi:10.1080/14686996.2026.2619337)
Supplement: Supplemental Material [file TSTA_A_2619337_SM0880.docx]

Supplementary Information

Single DNA hairpin nanowire based on self-hybridization chain reaction for sensitive ATP detection

Fengyi Lin,^a,b^ Jing Liu,^b^ Yuxin Cheng,^a^ Min Li,^a^ Hong Zhang,^c^ Cuisong Zhou,^a^ Yong Guo,^a^ Dan Xiao,^a^ Peng Mi^b,*^ and Jianyuan Dai^a,*^

^a^ College of Chemistry, Sichuan University, Chengdu, 610064, China.

^b^ Department of Radiology, Huaxi MR Research Center (HMRRC) and State Key Laboratory of Biotherapy, West China Hospital, Sichuan University, Chengdu, 610041, China.

^c^ Hospital of Chengdu University of Traditional Chinese Medicine, 37 Shi-er-qiao Road, Jinniu District, Chengdu, Sichuan, 610075, China

^*^Corresponding Author:

Peng Mi; Mailing address: State Key Laboratory of Biotherapy, West China Hospital, Sichuan University, Chengdu 610041, China; Telephone number: +86-19983137953; E-mail: [mi@scu.edu.cn](mailto:mi@scu.edu.cn);

Jianyuan Dai; Mailing address: College of Chemistry, Sichuan University, Chengdu 610064, China; Telephone number: +86-18380216833; E-mail: [daijy@scu.edu.cn](mailto:daijy@scu.edu.cn)

Table of Contents

[**Supplementary Tables** 2](#_Toc212474665)

[**Supplementary Figures** 8](#_Toc212474666)

[**References** 24](#_Toc212474667)

**Supplementary Tables**

**Table S1** The DNA sequences used in the research. *

| **DNA name** | **DNA sequence (5’ to 3’)** |
| --- | --- |
| H | TTTTTTGACCATGCGCGCAT-(FAM)-GGTCAAAAAAAAAAAAGACCAT-(BHQ1)-GCGCGCATGGTCTTTTTT |
| I | AAAAAAGACCATGCGCGCATGGTCAAAAAA |
| I_5A_ | AAAAAAGACCATGCGCGCATGGTCAAAAA |
| I_4A_ | AAAAAAGACCATGCGCGCATGGTCAAAA |
| I_3A_ | AAAAAAGACCATGCGCGCATGGTCAAA |
| I_2A_ | AAAAAAGACCATGCGCGCATGGTCAA |
| I_1A_ | AAAAAAGACCATGCGCGCATGGTCA |
| I_0A_ | AAAAAAGACCATGCGCGCATGGTC |
| Mismatched I | AAAAAAGACCATGTGCGCATGGTCAAAAAA |
| H1 | TTAACCCACGCCGAAT-(FAM)-CCTAGACTCAAAGTAGTCTAGGAT-(BHQ1)-TCGGCGTG |
| H2 | AGTCTAGGATTCGGCGTGGGTTAACACGCCGAATCCTAGACTACTTTG |
| I_HCR_ | AGTCTAGGATTCGGCGTGGGTTAA |
| Mismatched I_HCR_ | AGTCTAGTATTCGGCGTGGGTTAA |
| Trigger-Aptamer (TA) | AAAAAAGACCATGCGCGCATGGTCAAAAAAACCTGGGGGAGTATTGCGGAGGAAGGT |
| TA_1M_ | AAAAAAGACCATGCGCGCATGGTCAAAAAAACCTGGGGGAGTATTGCGTAGGAAGGT |
| TA_2M_ | AAAAAAGACCATGCGCGCATGGTCAAAAAAACCTGGGGGAGTATTGCGTATGAAGGT |
| Blocking probe (BP) | CCCAGGTTTTTTTGA |
| BP-3’-1 | CCCAGGTTTTTTTG |
| BP-3’+1 | CCCAGGTTTTTTTGAC |
| BP-3’+2 | CCCAGGTTTTTTTGACC |

* The red sequences are indicating those bases were changed compared to the determined sequence; The purple sequences represent trigger DNA; The green sequence in TA is ATP-binding aptamer.

**Table S2** Quantitative calculation of gray values and relative yields of each lane in gel electrophoresis image (Figure 1D) of the SHCR nanowires.

| **Lane** | **1** | **2** | **3** | **4** | **5** | **6** | **7** | **8** | **9** |
| --- | --- | --- | --- | --- | --- | --- | --- | --- | --- |
| Gray value of H hairpin dimer | 158685 | 150588 | 128126 | 73321 | 72166 | 79315 | 125828 | 174776 | 218397 |
| Gray value of H by-product | 134952 | 137289 | 137490 | 136829 | 135830 | 135412 | 136300 | 133495 | 134387 |
| Gray value of the SHCR nanowires | 566231 | 590837 | 694987 | 797342 | 819848 | 863282 | 919257 | 891917 | 834049 |
| Total gray value of the SHCR nanowires | 859868 | 878714 | 960603 | 1007492 | 1027844 | 1078009 | 1181385 | 1200188 | 1186833 |
| Relative yield of the SHCR nanowires (including by-product) | 0% | 7.87% | 32.6% | 52.4% | 54.9% | 58.0% | 57.4% | 51.4% | 43.2% |
| Relative yield of the SHCR nanowires (without by-product) | 0% | 14.0% | 50.1% | 75.9% | 77.8% | 78.9% | 73.7% | 65.1% | 55.1% |

**Table S3** Comparison of kinetic parameters and signal-to-noise ratio between the HCR and SHCR nanowires.

|  | **HCR** | **SHCR** |
| --- | --- | --- |
| Reaction time to plateau | 100 min | 60 min |
| Maximum background leakage rate (*V_l,max_*)* | 6.36×10^-11^ M·s^-1^ | 9.03×10^-11^ M·s^-1^ |
| Leakage rate constant (*k_l_*) | 2469.78 M^-1^·s^-1^ | 3952.22 M^-1^·s^-1^ |
| Maximum reaction rate (*V_r,max_*)* | 1.72×10^-10^ M·s^-1^ | 1.31×10^-10^ M·s^-1^ |
| Forward rate constant (*k_on,Toe_*) | 1908.29 M^-1^·s^-1^ | 510.56 M^-1^·s^-1^ |
| Reverse rate constant (*k_off,Toe_*) | 8.28×10^-2^ s^-1^ | 2.22×10^-2^ s^-1^ |
| Branch migration (*k_b_*) | 1.23 s^-1^ | 1.23 s^-1^ |
| Signal-to-noise ratio** | 51.6 | 102.7 |

* *V_l,max_* and *V_r,max_* are the peaks of the corresponding reaction rate curve (Figure S7A, Figure S7B), namely the maximum values [1].

** Signal-to-noise ratios were calculated from the linear portion of each fluorescence curve (Figure 2B) [2].

**Table S4** Standard free energy (Δ*G*) for each reaction process of HCR and SHCR calculated by OligoAnalyzer Tool (Integrated DNA Technologies, Inc.).

| **Reaction process** | **Reaction equation** | **Δ*G*/kcal·mol^-1^** |
| --- | --- | --- |
| Background | HCR: H_1_ + H_2_ → H_1_-H_2_ | 2.58 |
|  | SHCR: H + H → H-H | -5.29 |
| Initiating | HCR: I_HCR_ + H_1_ → I_HCR_-H_1_ | -23.42 |
|  | SHCR: I + H → I-H | -13.58 |
| Circuit | HCR: I_HCR_-H_1_ + H_2_ → I_HCR_-H_1_-H_2_ | -22.29 |
|  | SHCR: I-H + H → I-H-H | -34.01 |

**Table S5** Summary and comparison of different sensors for ATP detection.

| **Strategy** | **Signal readout** | **Linear range** | **Limit of detection** | **Ref.** |
| --- | --- | --- | --- | --- |
| Graphene oxide aptamer Beacon | Fluorescence | 0.125 ~ 2 mM | 19 μM | [3] |
| Conjugated polymer | Fluorescence | 0 ~ 180 mM | 2.5 μM | [4] |
| DNA triangular prism | Fluorescence | 0.03 ~ 2 mM | 30 μM | [5] |
| Carbon dots | Colorimetry | 0.05 ~ 0.4 mM | 9 μM | [6] |
| DNA nanolantern | Fluorescence | 0 ~ 1 mM | 8.3 μM | [7] |
| **SHCR** | **Fluorescence** | **0 ~ 150 μM** | **0.368 μM** | **This work** |

**Table S6** Quantitative evaluation of ATP concentration based on the fluorescence intensity in Figure 5B.

|  | **Relative fluorescence unit** | **ATP concentration measured by the SHCR nanowire*** | **ATP concentration in the original supernatant**** | **RSD%** |
| --- | --- | --- | --- | --- |
| 4T1 cell + SHCR | 12405 | 2.3 μM | 46 μM | 5.02 |
| 4T1 cell + X-ray + SHCR | 40201 | 296 μM | 5.92 mM | 5.24 |
| BxPC-3 cell + SHCR | 13157 | 5.7 μM | 114 μM | 8.35 |
| BxPC-3 cell + X-ray + SHCR | 42591 | 419 μM | 8.38 mM | 2.95 |

* The sample solution with a final volume of 100 μL was prepared by combining 95 μL of the SHCR nanowire with 5 μL supernatant, and the relative fluorescence unit of the SHCR nanowire was measured and fitted to the curve shown in Figure S14 for determining ATP concentration.

** ATP concentrations in the original supernatant were calculated according to the ATP concentration measured by the SHCR nanowire, where the value of the former is 20 times greater than that of the latter.

**Supplementary Figures**


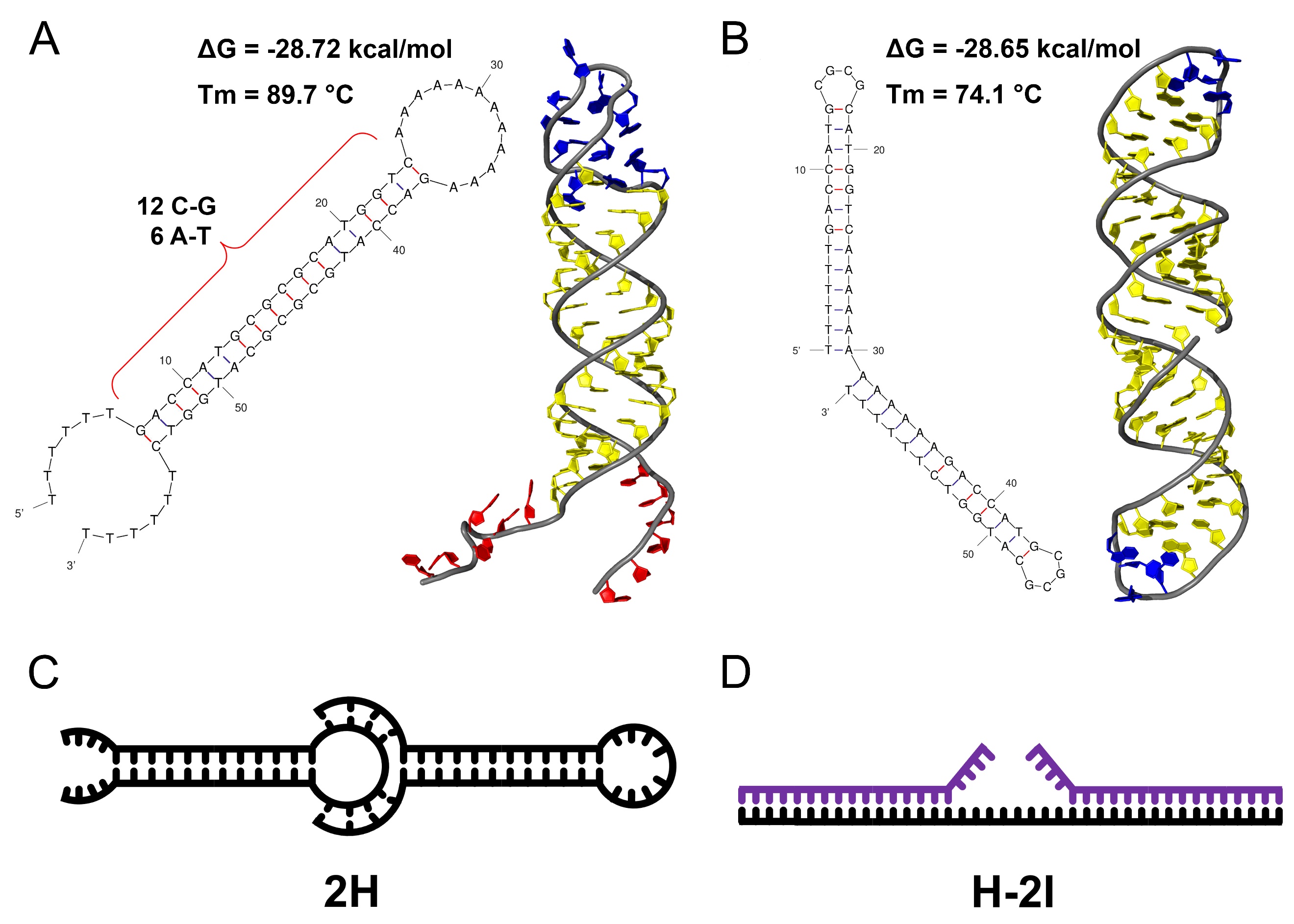


**Figure S1** (A) The secondary structure (left) and tertiary structure (right) of H principal product. (B) The secondary structure (left) and tertiary structure (right) of the H by-product. In the tertiary structure, red is the toehold, yellow is the stem, and blue is the loop. (C) The H hairpin dimer structure. (D) Two I was combined with H to form an H-2I structure.


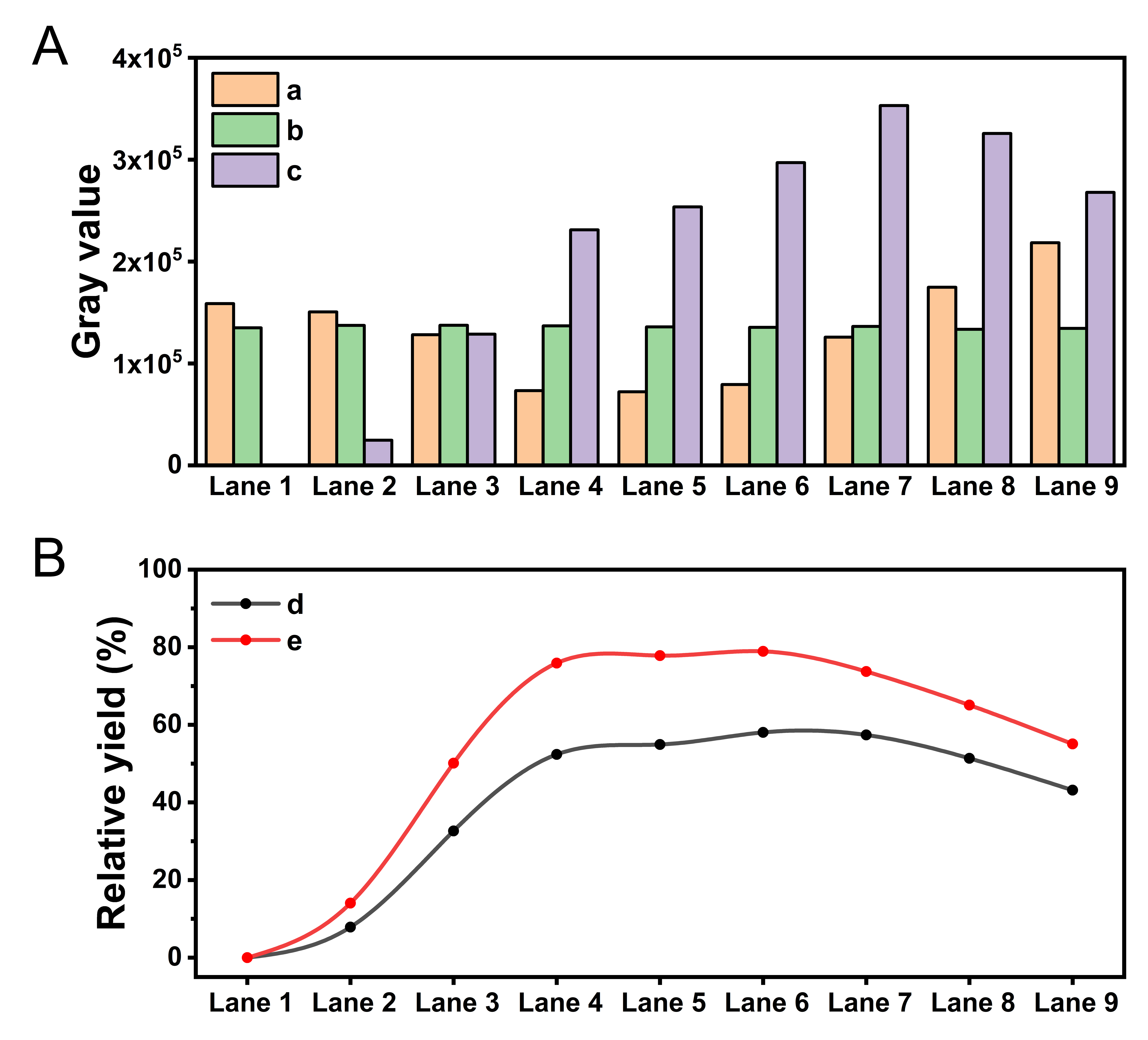


**Figure S2** (A) Gray values of each lane in gel electrophoresis image (Figure 1D): (a) gray values of the band corresponding to H hairpin dimer, (b) gray values of the band corresponding to by-product, (c) the difference in gray values between the band area of the SHCR nanowires and the same area in lane 1. (B) Relative yields of the SHCR nanowires: (d) the relative yield including by-product, (e) the relative yield without by-product.


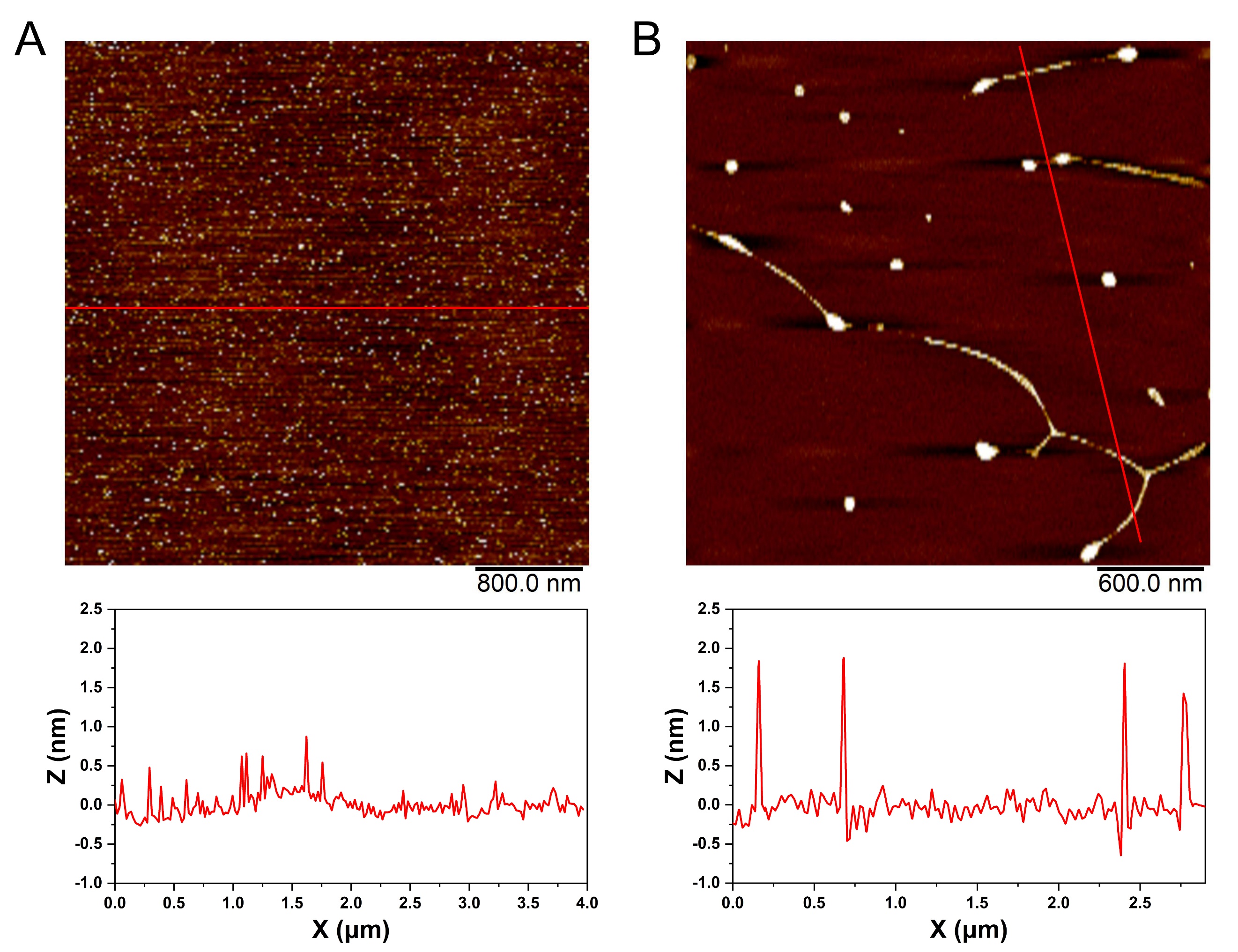


**Figure S3** AFM images and cross-section analysis of (A) before and (B) after adding I into the SHCR nanowire.


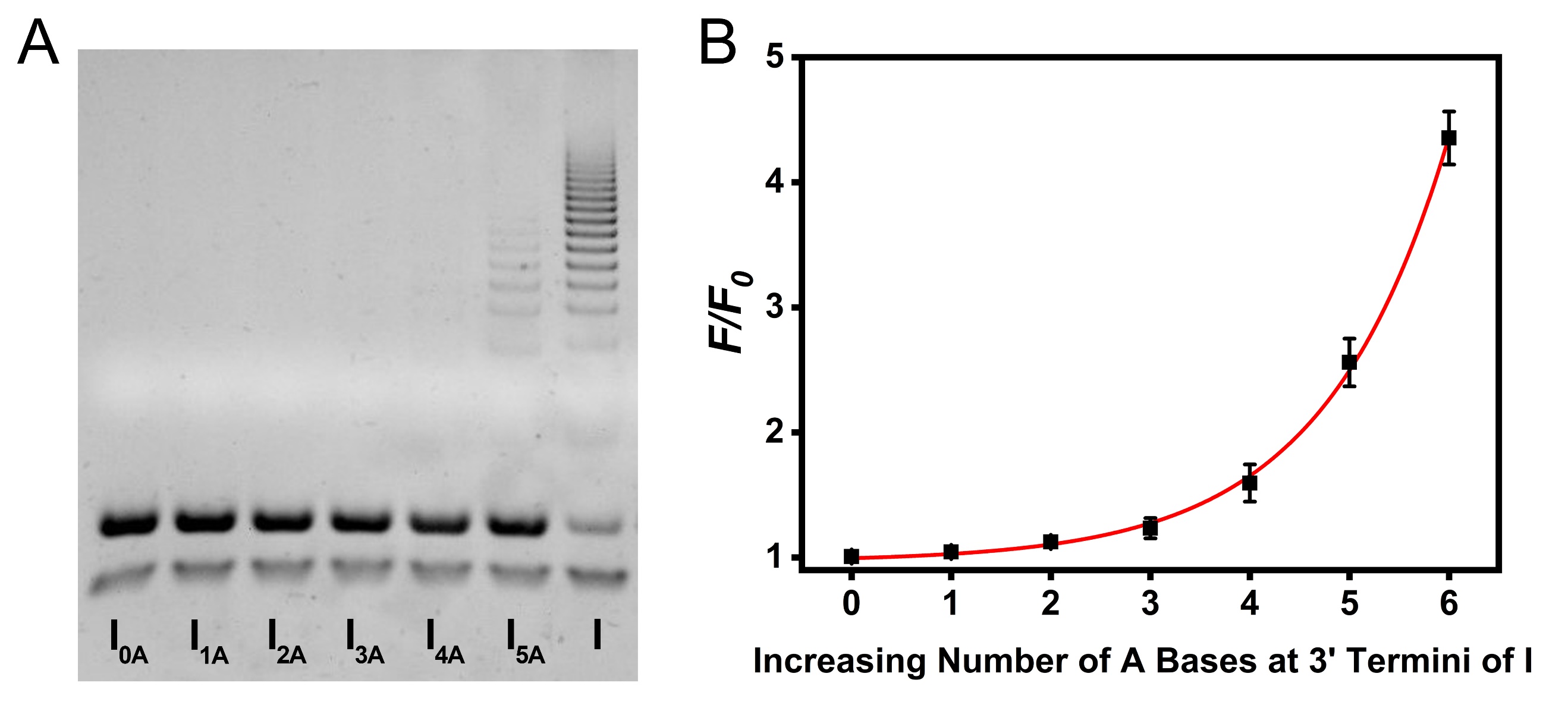


**Figure S4** (A) Gel electrophoresis image of H reacting with different I (I_0A_, I_1A_, I_2A_, I_3A_, I_4A_, I_5A_ and I). The subscript number of "I" represents the number of A bases at 3’ end of I, and I has 6 A bases at 3' end. (B) Relative fluorescence intensity changes with the increasing numbers of A bases at 3’ end of I.


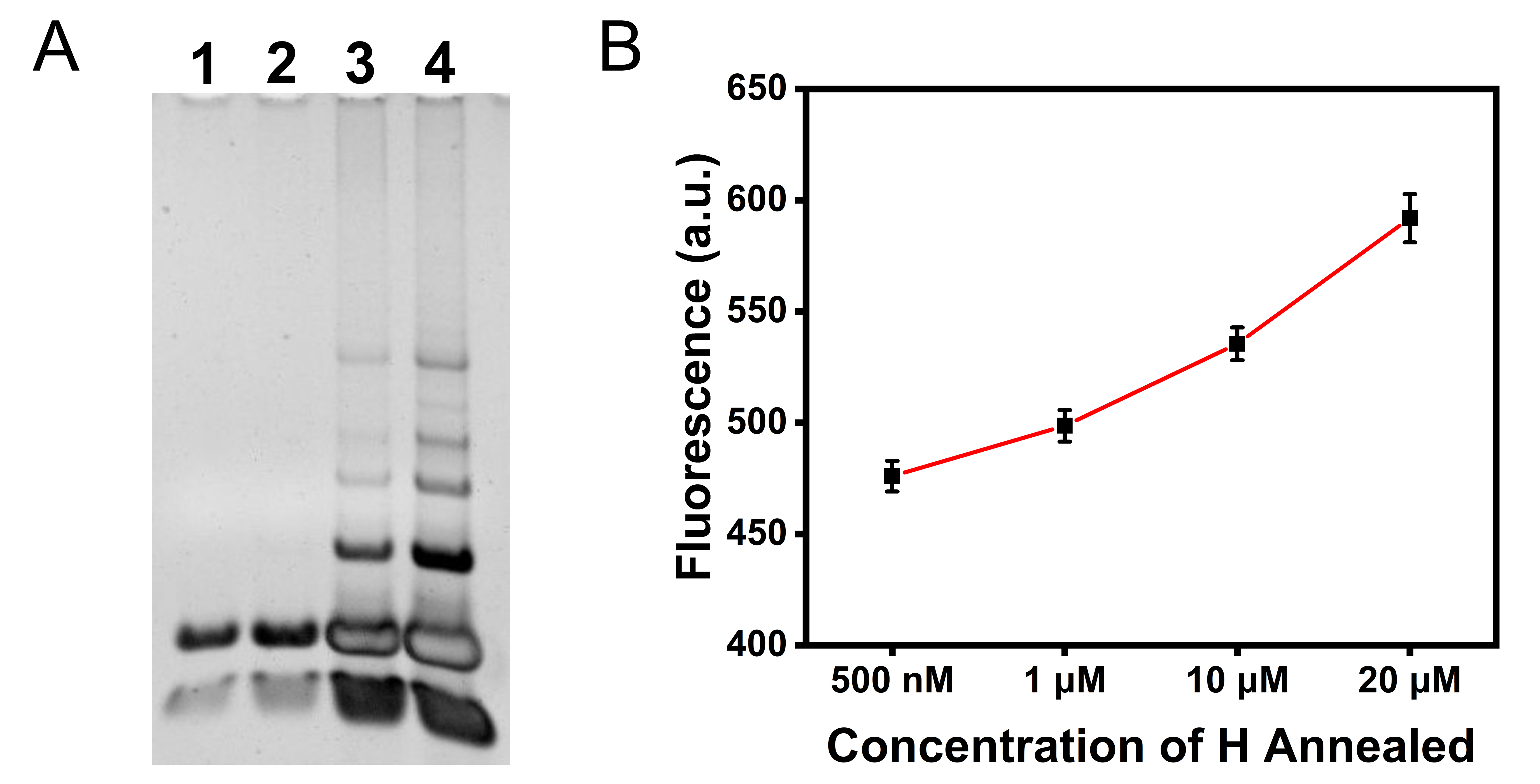


**Figure S5** (A) Gel electrophoresis image of annealed H with different concentrations. Lane 1: 500 nM; Lane 2: 1 μM; Lane 3: 10 μM; Lane 4: 20 μM. (B) Fluorescence intensity of annealed H with different concentrations. The samples were diluted into the same concentration (500 nM) when fluorescence intensity was measured.

**
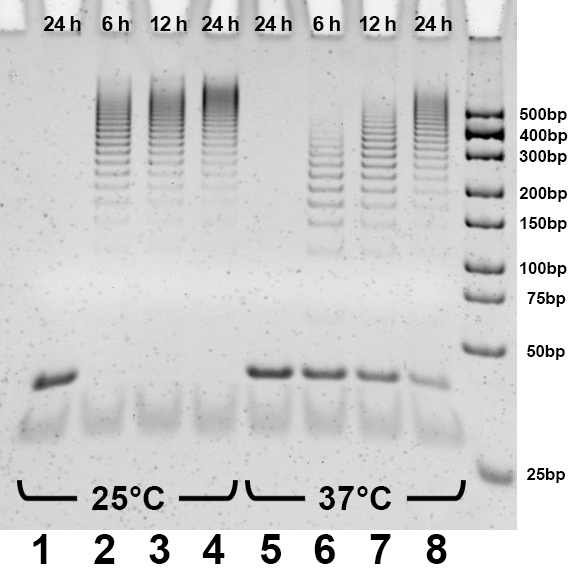
**

**Figure S6** Gel electrophoresis image of the SHCR nanowires in the absence (lane 1 and lane 5) and presence (lane 2-4 and lane 6-8) of 50 nM I at different temperatures (25 °C and 37 °C) for different reaction times (6 h, 12 h and 24 h).

**
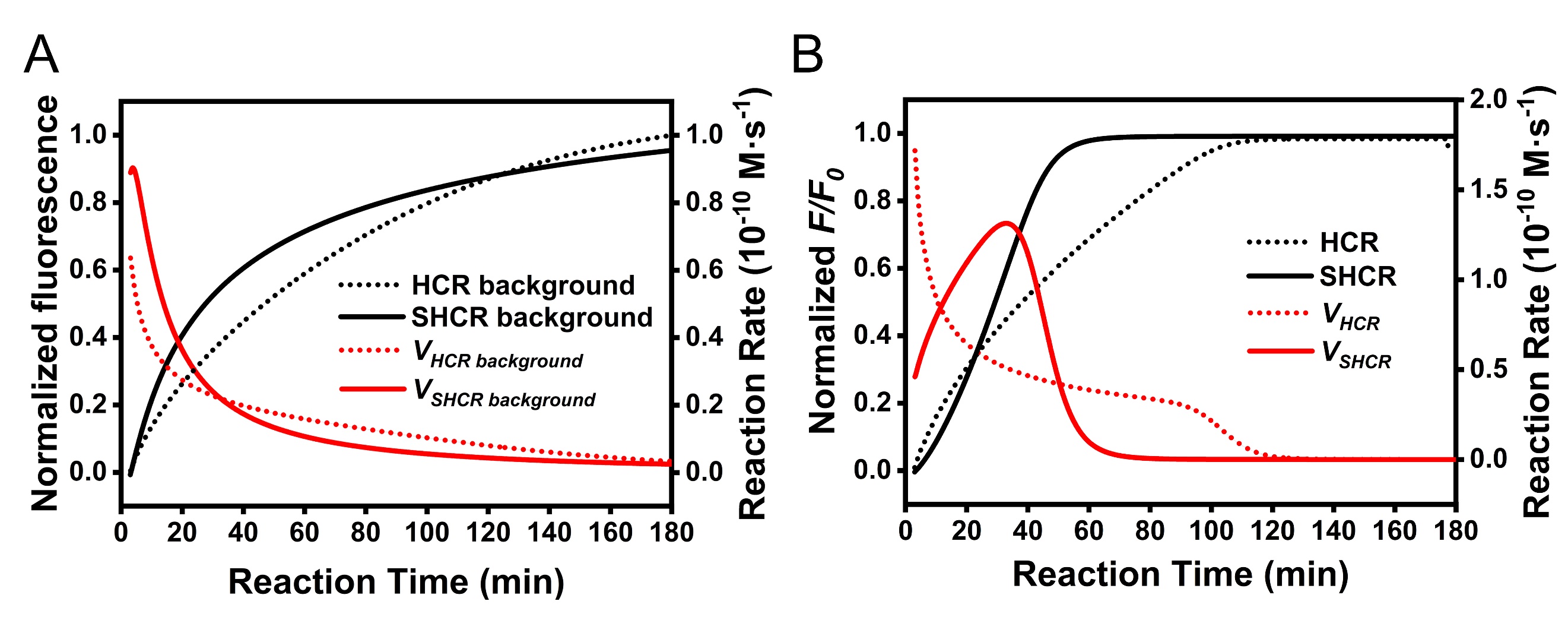
**

**Figure S7** (A) The fitting curve (black line) and background leakage rate curve (red line) of the normalized fluorescence values of the HCR and SHCR nanowires. (B) The fitting curve (black line) and reaction rate curve (red line) of the normalized relative fluorescence intensity of HCR and SHCR. H1, H2, H, and bare H Concentration: 150 nM. Concentrations of I_HCR_ and I: 50 nM.

**
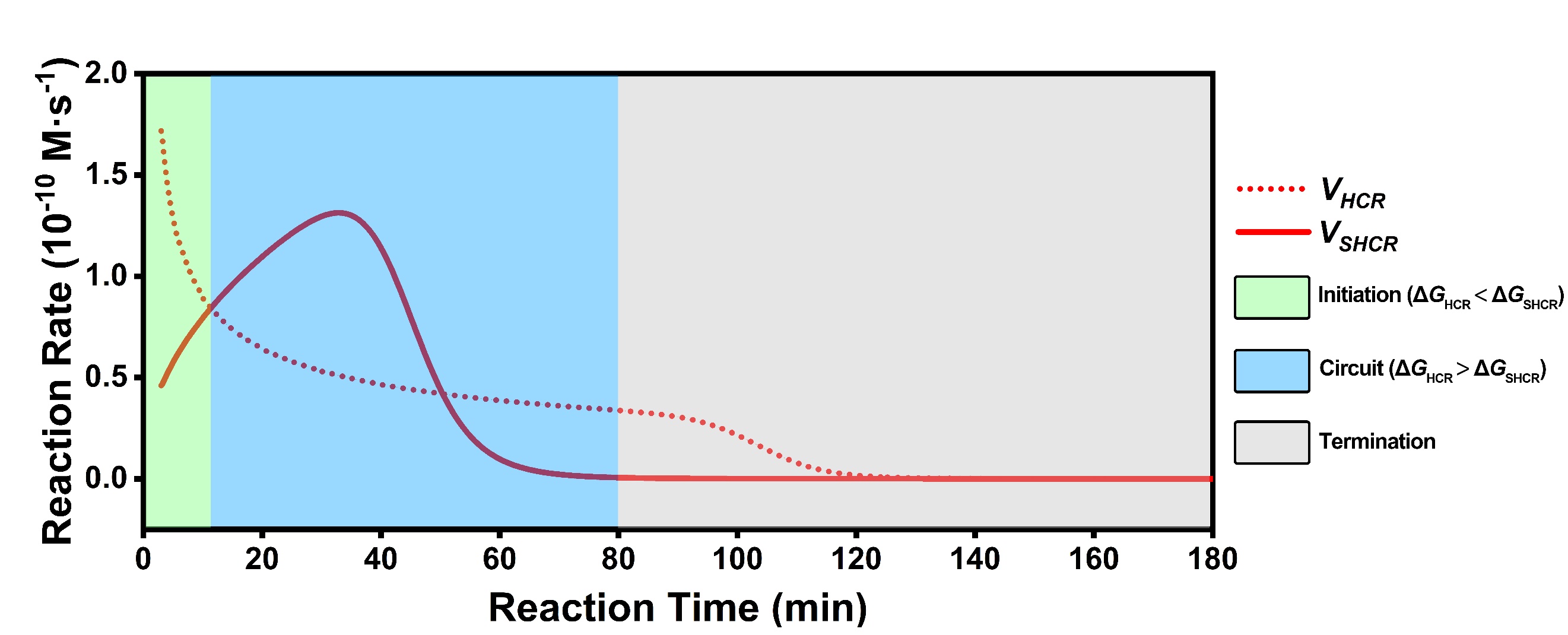
**

**Figure S8** The reaction rate of HCR and SHCR from Figure S7B correspond to the reaction process according to the comparison of standard free energy variations (Δ*G*_HCR_ and Δ*G*_SHCR_).


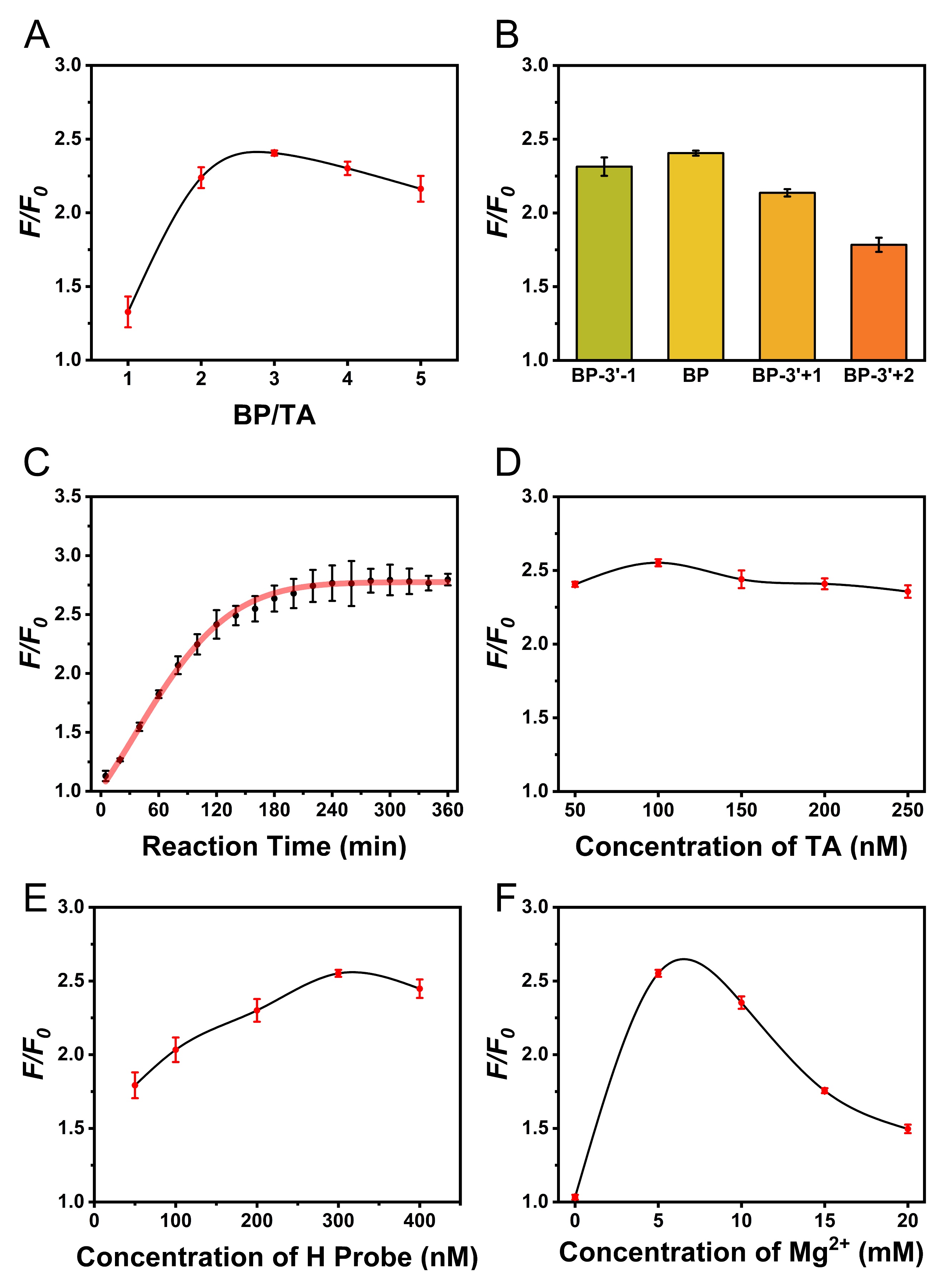


**Figure S9** Optimization of the SHCR nanowire for ATP detection. (A) Ratios of BP to TA (BP/TA), (B) the length of BP, (C) reaction time, concentrations of (D) TA, (E) H and (F) Mg^2+^ in Tris buffer.


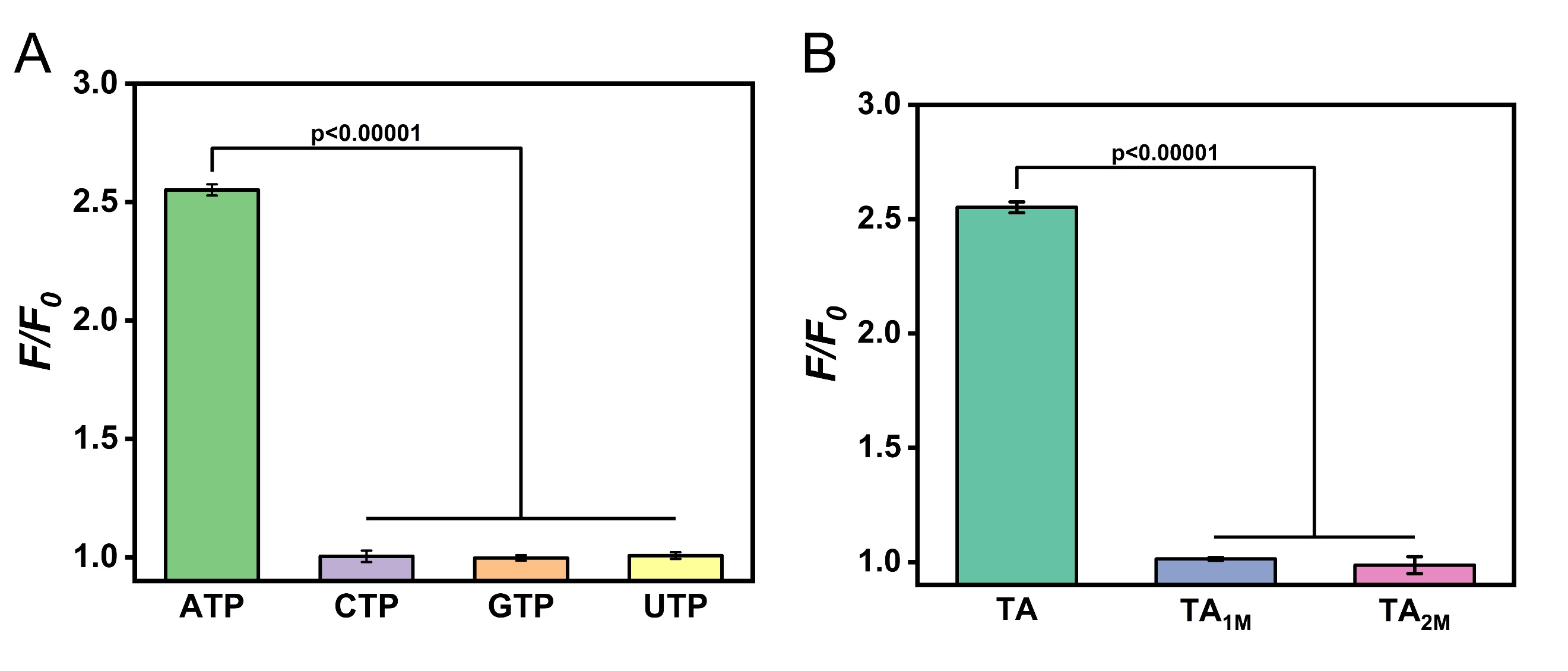


**Figure S10** (A) The effect of ATP analogous molecules on the selectivity. The concentrations of ATP, CTP, GTP and UTP: 100 μM. (B) The effect of mutated TA on the SHCR nanowire. Data are shown as mean values ± s.d. (n=3). *p*-values were determined by one-way ANOVA with Tukey’s post hoc test.


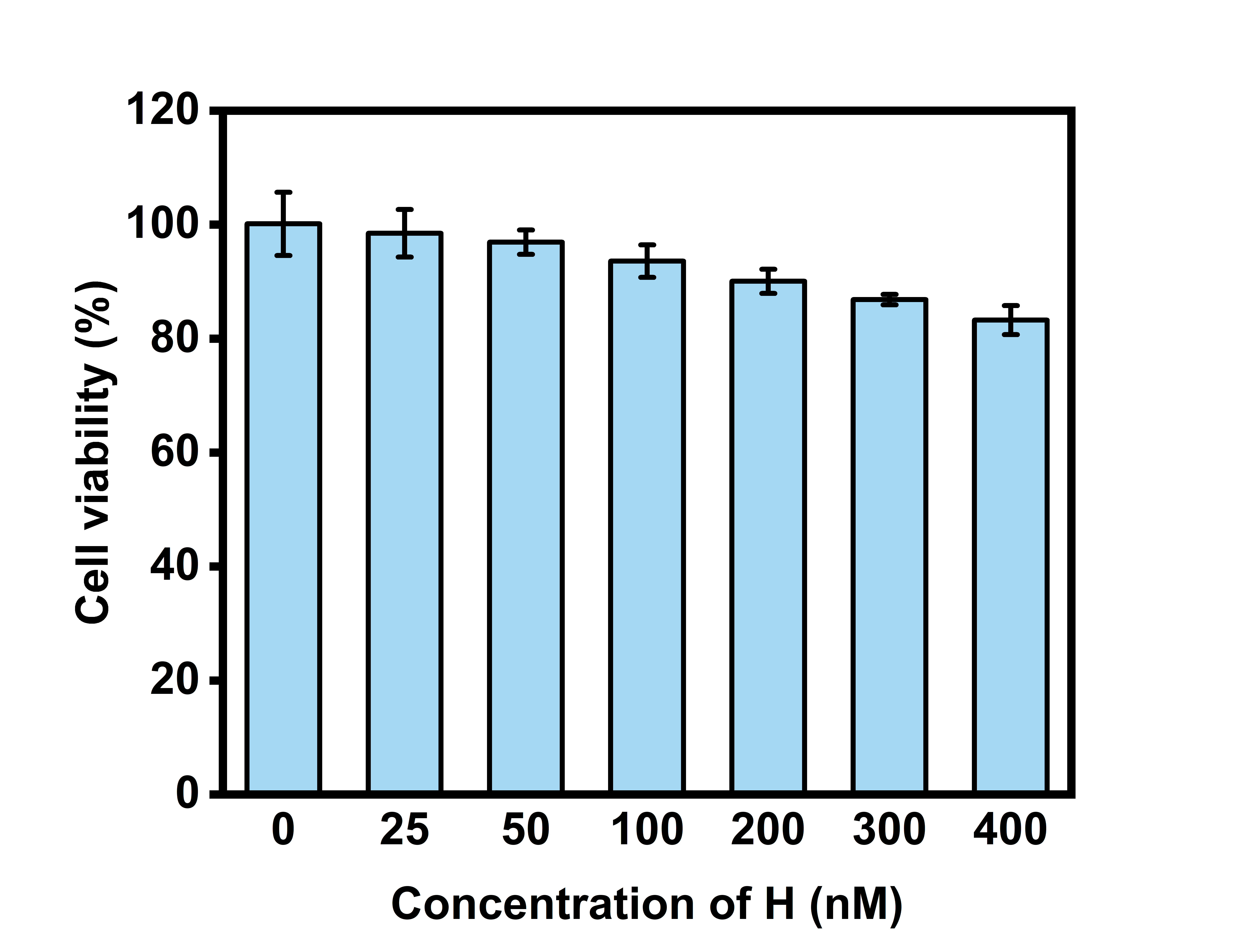


**Figure S11** Cell viability of 4T1 cells incubated with different concentrations of H (0, 25, 50, 100, 200, 300, 400 nM) for 24 h, respectively. In the SHCR nanowire, the concentration of TA-BP is one-third that of H.


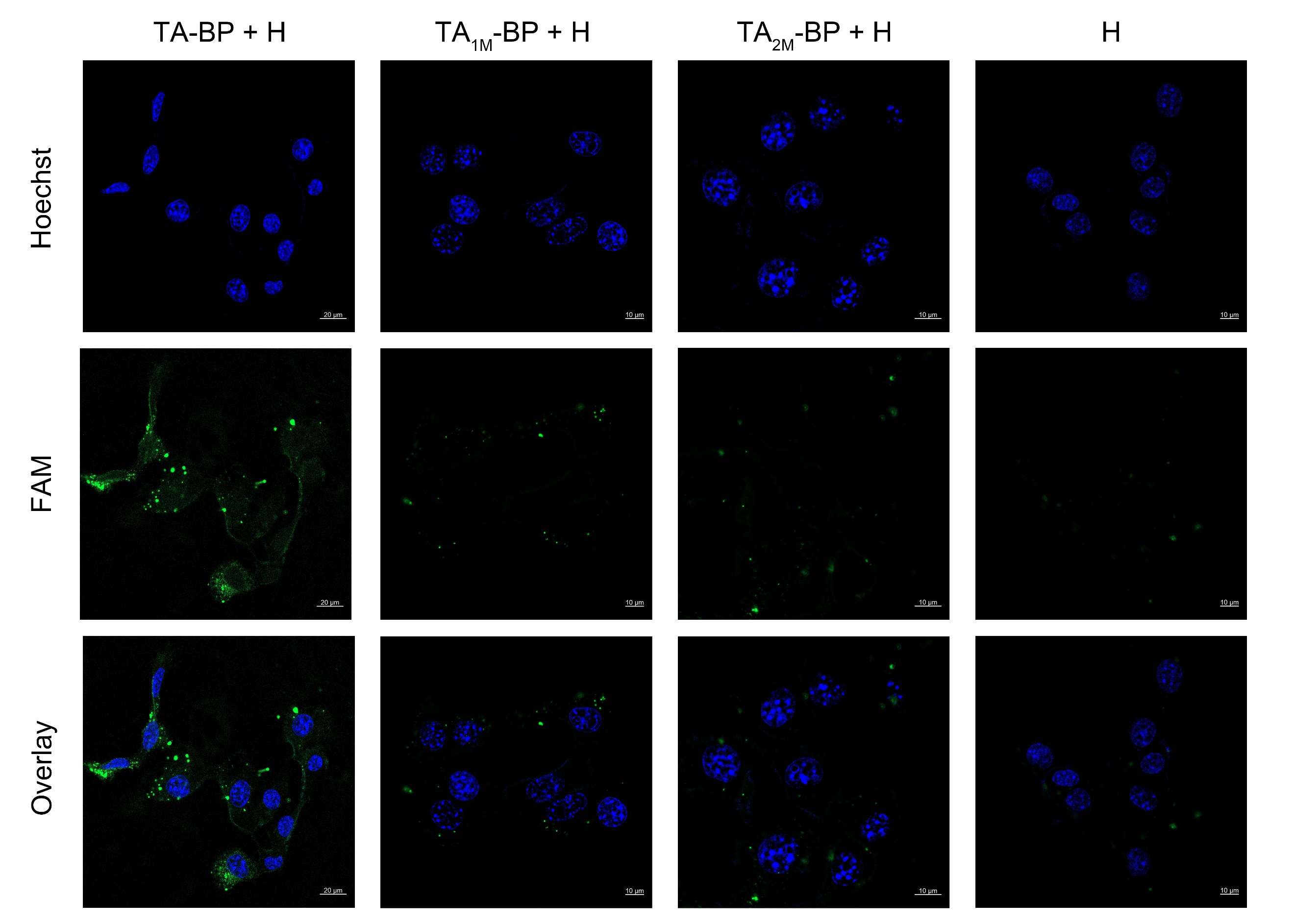


**Figure S12** CLSM images of ATP in different systems (TA-BP+H, TA_1M_-BP+H, TA_2M_-BP+H, and H) in 4T1 cells.


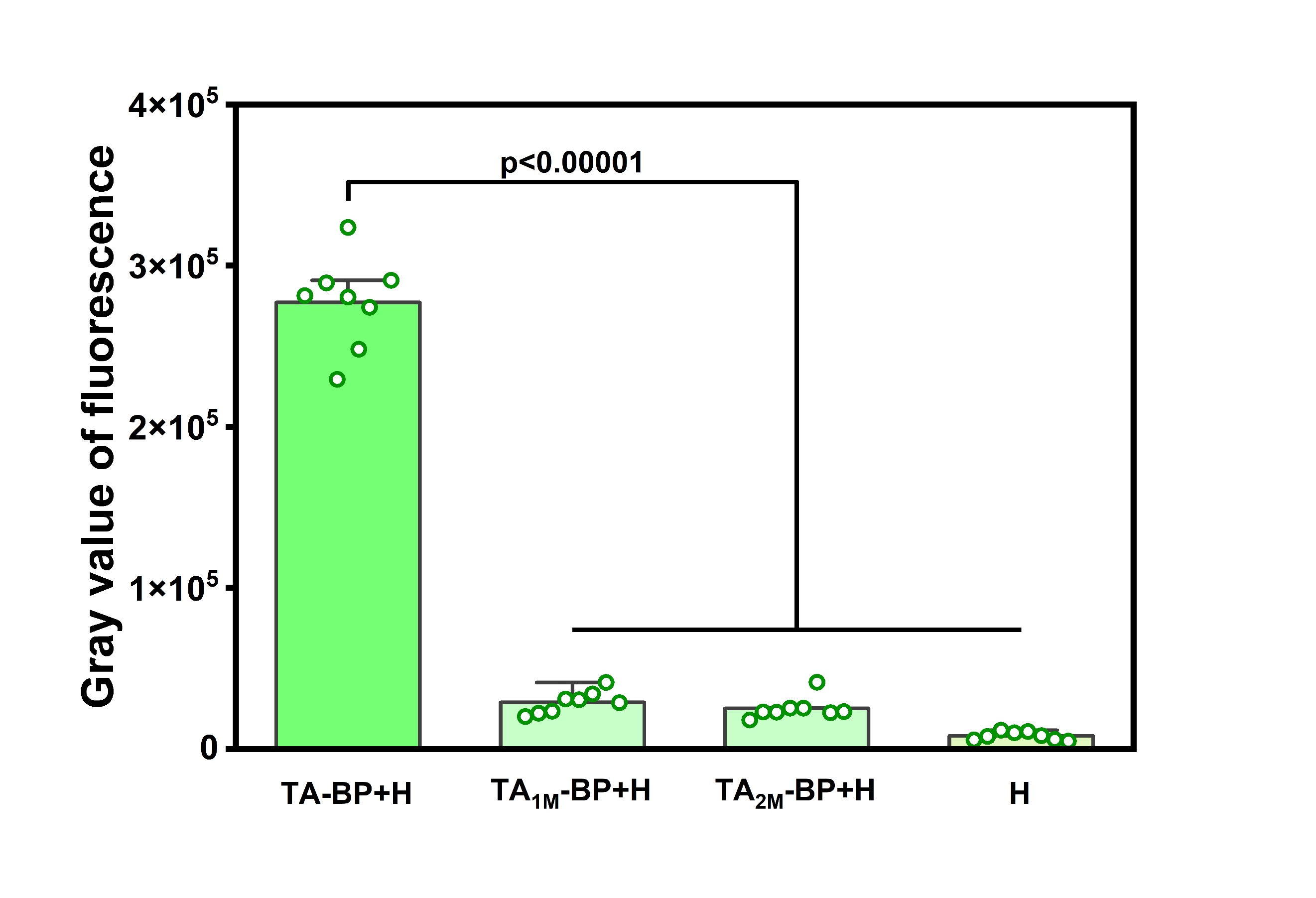


**Figure S13** The statistical analysis of the gray value of fluorescence intensity. Data are shown as mean values ± s.d. (n=8). *p*-values were determined by one-way ANOVA with Tukey’s post hoc test.


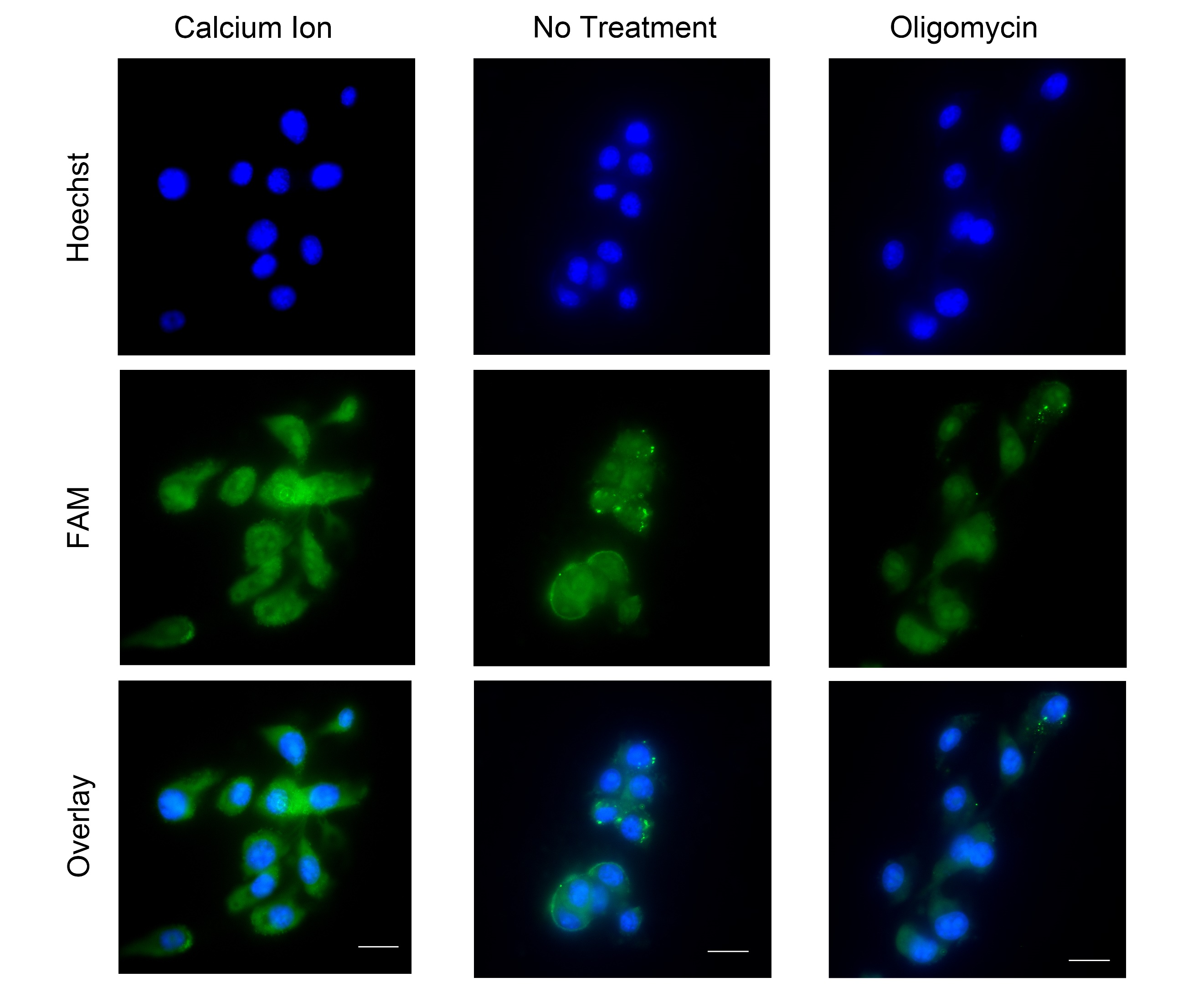


**Figure S14** CLSM images of ATP in 4T1 cells under different treatments. All scale bars are 20 μm.


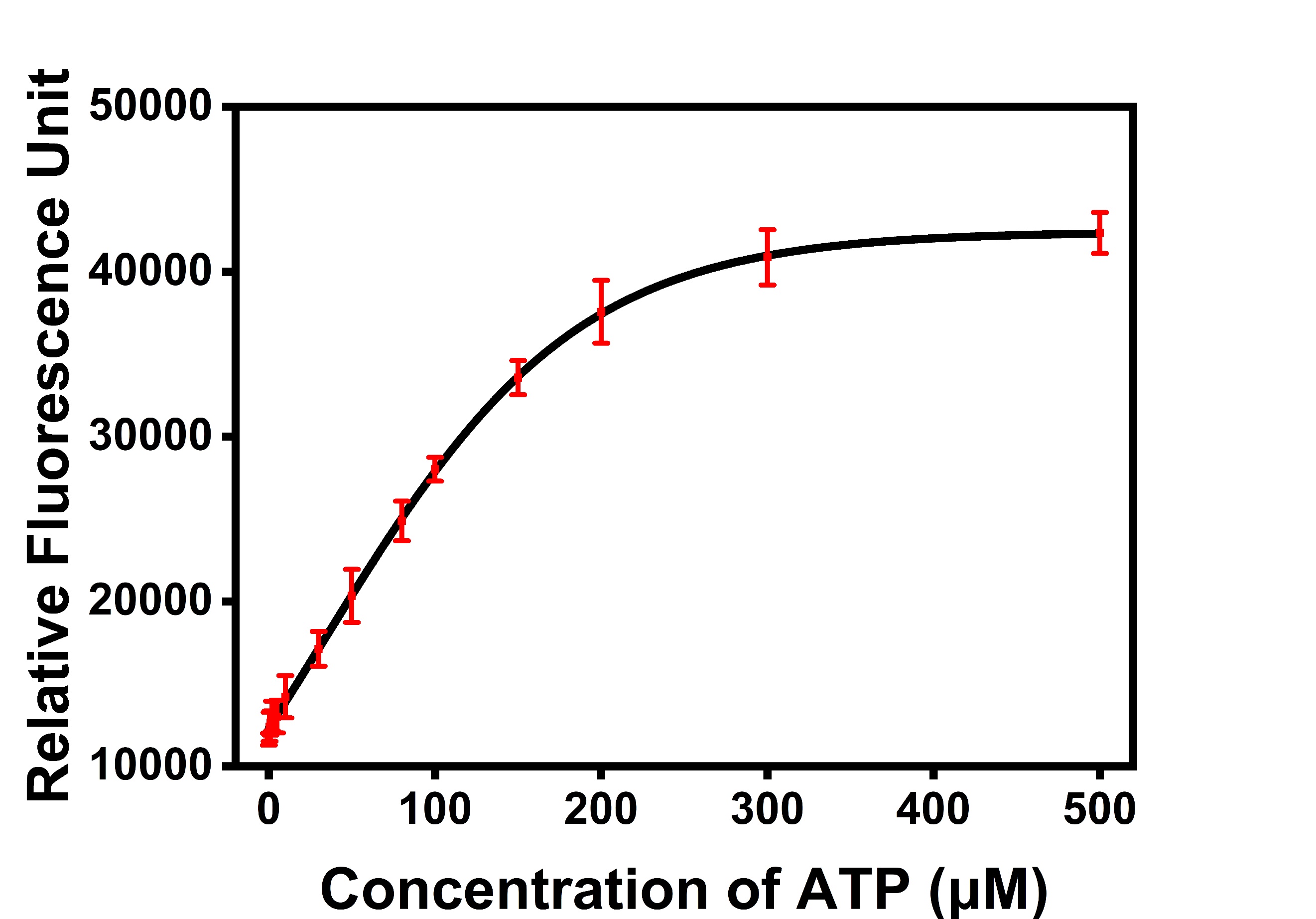


**Figure S15** Relative fluorescence unit of the SHCR nanowire in responding to different ATP concentrations. ATP concentration: 0, 0.1, 0.2, 0.5, 1.0, 1.5, 2.0, 5.0, 10, 30, 50, 80, 100, 150, 200, 300 and 500 μM.


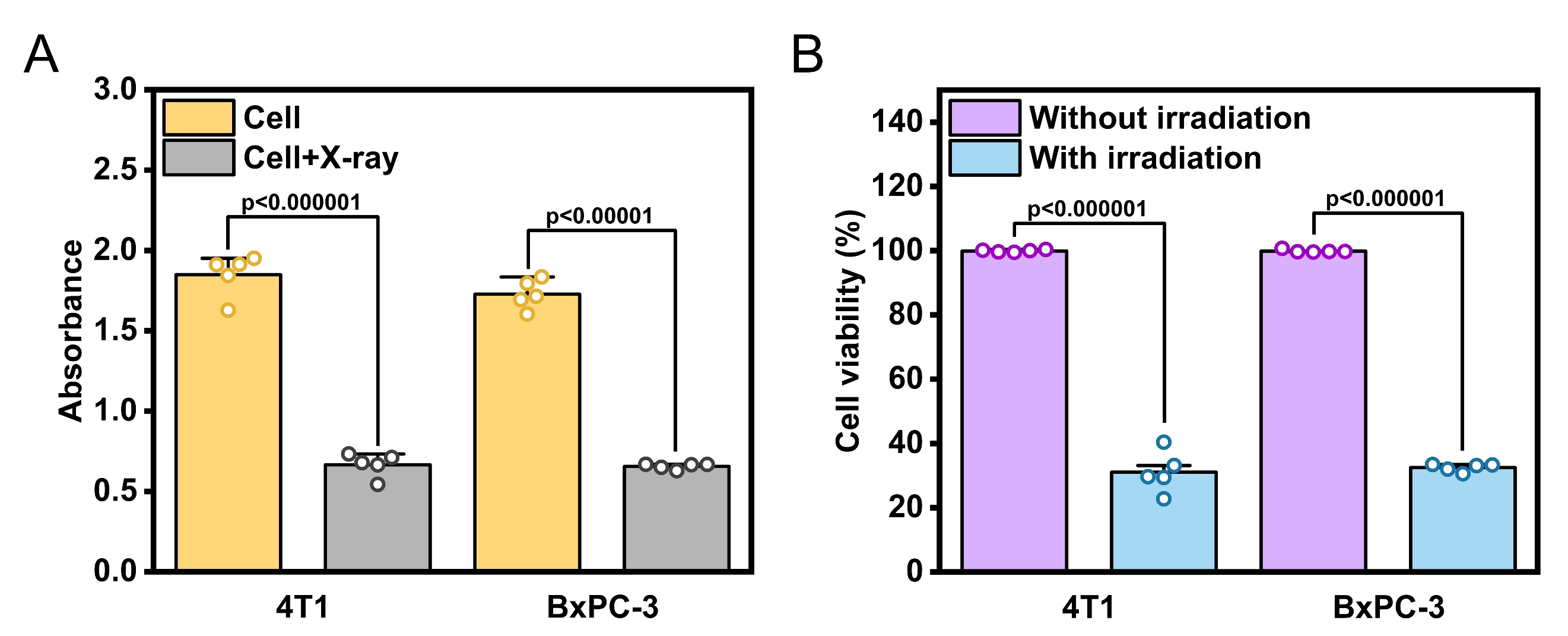


**Figure S16** (A) Absorbance signal of 4T1 and BxPC-3 cells by CCK-8 before and after X-ray irradiation. (B) Cell viability of 4T1 and BxPC-3 cells before and after X-ray irradiation was calculated using formula (9). Data are shown as mean values ± s.d. (n=5). *p*-values were determined by two-tailed Student’s *t* test.

**References**

[1] Wu L, Wang GA, Li F. Plug-and-Play Module for Reversible and Continuous Control of DNA Strand Displacement Kinetics. J Am Chem Soc. 2024;146(10): 6516. doi:10.1021/jacs.3c09242.

[2] Jiang YS, Bhadra S, Li B, et al. Mismatches Improve the Performance of Strand-Displacement Nucleic Acid Circuits. Angew Chem Int Ed. 2014;53(7): 1845. doi:10.1002/anie.201307418.

[3] Liu Z, Chen S, Liu B, et al. Intracellular Detection of ATP Using an Aptamer Beacon Covalently Linked to Graphene Oxide Resisting Nonspecific Probe Displacement. Anal Chem. 2014;86(24): 12229. doi:10.1021/ac503358m.

[4] Zhao Q, Zhang Z, Tang Y. A New Conjugated Polymer-Based Combination Probe for ATP Detection Using a Multisite-Binding and Fret Strategy. Chem Commun. 2017;53(68): 9414. doi:10.1039/c7cc04293k.

[5] Zheng X, Peng R, Jiang X, et al. Fluorescence Resonance Energy Transfer-Based DNA Nanoprism with a Split Aptamer for Adenosine Triphosphate Sensing in Living Cells. Anal Chem. 2017;89(20): 10941. doi:10.1021/acs.analchem.7b02763.

[6] Geng X, Sun Y, Guo Y, et al. Fluorescent Carbon Dots for in Situ Monitoring of Lysosomal ATP Levels. Anal Chem. 2020;92(11): 7940. doi:10.1021/acs.analchem.0c01335.

[7] Wang YX, Wang DX, Ma JY, et al. DNA Nanolantern-Based Split Aptamer Probes for in Situ ATP Imaging in Living Cells and Lighting up Mitochondria. Analyst. 2021;146(8): 2600. doi:10.1039/d1an00275a.
